# Activated cholesterol metabolism is integral for innate macrophage responses by amplifying Myd88 signaling

**DOI:** 10.1172/jci.insight.138539

**Published:** 2022-11-22

**Authors:** Sumio Hayakawa, Atsushi Tamura, Nikita Nikiforov, Hiroyuki Koike, Fujimi Kudo, Yinglan Cheng, Takuro Miyazaki, Marina Kubekina, Tatiana V. Kirichenko, Alexander N. Orekhov, Nobuhiko Yui, Ichiro Manabe, Yumiko Oishi

**Affiliations:** 1 Department of Biochemistry and Molecular Biology, Graduate School of Medicine, Nippon Medical School, Tokyo, Japan.; 2 Department of Organic Biomaterials, Institute of Biomaterials and Bioengineering, Tokyo Medical and Dental University, Tokyo, Japan.; 3 National Medical Research Center of Cardiology, Institute of Experimental Cardiology, Moscow, Russia.; 4 Institute of Gene Biology, Centre of Collective Usage, Moscow, Russia.; 5 Laboratory of Angiopathology, Institute of General Pathology and Pathophysiology, Moscow, Russia.; 6 Department of Systems Medicine, Chiba University Graduate School of Medicine, Chiba, Japan.; 7 Department of Biochemistry, Showa University School of Medicine, Tokyo, Japan.; 8 Institute for Atherosclerosis Research, Moscow, Russia.

**Keywords:** Inflammation, Vascular Biology, Innate immunity

## Abstract

Recent studies have shown that cellular metabolism is tightly linked to the regulation of immune cells. Here, we show that activation of cholesterol metabolism, involving cholesterol uptake, synthesis, and autophagy/lipophagy, is integral to innate immune responses in macrophages. In particular, cholesterol accumulation within endosomes and lysosomes is a hallmark of the cellular cholesterol dynamics elicited by Toll-like receptor 4 activation and is required for amplification of myeloid differentiation primary response 88 (Myd88) signaling. Mechanistically, Myd88 binds cholesterol via its CLR recognition/interaction amino acid consensus domain, which promotes the protein’s self-oligomerization. Moreover, a novel supramolecular compound, polyrotaxane (PRX), inhibited Myd88‑dependent inflammatory macrophage activation by decreasing endolysosomal cholesterol via promotion of cholesterol trafficking and efflux. PRX activated liver X receptor, which led to upregulation of ATP binding cassette transporter A1, thereby promoting cholesterol efflux. PRX also inhibited atherogenesis in *Ldlr^–/–^* mice. In humans, cholesterol levels in circulating monocytes correlated positively with the severity of atherosclerosis. These findings demonstrate that dynamic changes in cholesterol metabolism are mechanistically linked to Myd88‑dependent inflammatory programs in macrophages and support the notion that cellular cholesterol metabolism is integral to innate activation of macrophages and is a potential therapeutic and diagnostic target for inflammatory diseases.

## Introduction

Recent studies in immunometabolism have shed light on the complex and intricate links between immunity and metabolism at the cell, tissue, and system levels (1, 2). At the cellular level, dynamic regulation of metabolism is tightly linked to immune cell activation. For instance, cellular immunometabolic mechanisms are important for proper immune responses to infection but also play a role in the immune exhaustion seen in cases of chronic infection and cancer (3). Although it remains underexplored, cellular immunometabolic regulation of immune cells may also contribute to the development of cardiovascular disease.

Atherosclerosis is a chronic inflammatory disease that remains the leading cause of cardiovascular diseases (4). Atherosclerotic plaques are characterized by extensive remodeling of the arterial wall, a feature of chronic inflammation, along with accumulation of lipids, particularly cholesterol. Macrophages are the major immune cell type within atherosclerotic plaques. Macrophages not only engulf modified LDL to become foam cells, a major cause of subendothelial lipid deposition, but also mediate vascular inflammation and remodeling (5, 6). Indeed, atherosclerotic plaque formation can be suppressed by inhibiting inflammatory activation of macrophages (7). Macrophages recognize molecules associated with pathogens and cell damage via pattern recognition receptors, such as Toll-like receptors (TLRs), which activate signals leading to transcription factors that control genes required for inflammatory responses. For instance, TLR4 recognizes lipopolysaccharide (LPS) and activates NF-κB in response. TLR4 appears to also recognize endogenous ligands and to contribute to atherogenesis (8).

Cholesterol is a crucial component of cell membranes and is essential for membrane trafficking and signaling and for cell proliferation (9, 10). All mammalian cells have complex mechanisms that govern cellular cholesterol homeostasis. Macrophages take up lipids in the forms of LDL, VLDL, and oxidized lipoproteins from the microenvironment via phagocytosis, macropinocytosis, and scavenger receptor-mediated pathways and by engulfing dying cells (11, 12). After the uptake, the lipoprotein-containing cargos are transported to early endosomes, where the molecules are sorted for recycling or degradation. Early endosomes mature into late endosomes, which then fuse with lysosomes (13). In addition, lipophagy, a specialized autophagy mediating lipid degradation, can deliver cholesterol from lipid droplets to lysosomes. Within the lysosomal lumen, cholesterol esters are hydrolyzed by acid lipase to generate free cholesterol (14). The cholesterol may then be delivered to other organelles, in part through membrane contact sites, as well as to the plasma membrane (14, 15). Niemann-Pick disease type C1 and C2 (NPC1 and NPC2) proteins play a crucial role in the export of cholesterol from lysosomes. Cholesterol transported to the ER membrane may turn off the transcriptional program for cholesterol biosynthesis as well as its uptake by sterol regulatory element–binding proteins (SREBPs) (16). Lysosomes are thus integral to the cellular processes involved in cholesterol uptake, trafficking, synthesis, and efflux. In that regard, earlier studies showed that, within foam cells in advanced atherosclerotic plaques, most cholesterol appears to be trapped within lysosomes (17) and that inhibition of lysosomal cholesterol efflux promotes atherosclerosis plaque formation (18). This suggests lysosomal cholesterol accumulation during atherogenesis is linked to macrophage function.

Excess cholesterol is either stored as cholesterol esters in cytoplasmic lipid droplets or excreted (19). Macrophages have multiple ways to protect against accumulation of toxic levels of free cholesterol, including increased esterification to form relatively inert cholesterol ester, increased phospholipid synthesis, and increased cholesterol efflux. Cholesterol efflux is mediated by scavenger receptor class B type 1 (SR-BI), ATP binding cassette transporter A1 (ABCA1), ATP binding cassette transporter G1 (ABCG1), and unmediated aqueous transfer (20, 21).

Accumulation of cellular free cholesterol induced by deletion of *Abca1* and/or *Abcg1* associates with enhanced inflammatory responses in macrophages due in part to increases in plasma membrane lipid rafts, which are rich in cholesterol and sphingolipids and serve as platforms for surface receptors, including TLR4 (22, 23). Excess cholesterol within lysosomes may lead to the generation of cholesterol crystals, which in turn activate NOD-like receptor family pyrin domain containing 3 inflammasomes (24, 25). Thus, dysregulation of cellular cholesterol homeostasis leads to pathological activation of macrophages. On the other hand, it has also been shown that cellular cholesterol levels are temporally changed after TLR4 activation (26, 27), which suggests cholesterol metabolism is actively regulated during innate activation of macrophages. We therefore hypothesized that cellular cholesterol metabolism is an essential component of macrophage activation programs. Here, we show that the activation of cellular cholesterol metabolism is important for inflammatory activation of monocytes/macrophages dependent on myeloid differentiation primary response 88 (Myd88) and is an attractive immunometabolic target for novel therapeutic strategies against atherosclerosis.

## Results

### Cellular cholesterol accumulates in response to TLR4 activation in macrophages.

**Figure 1:**
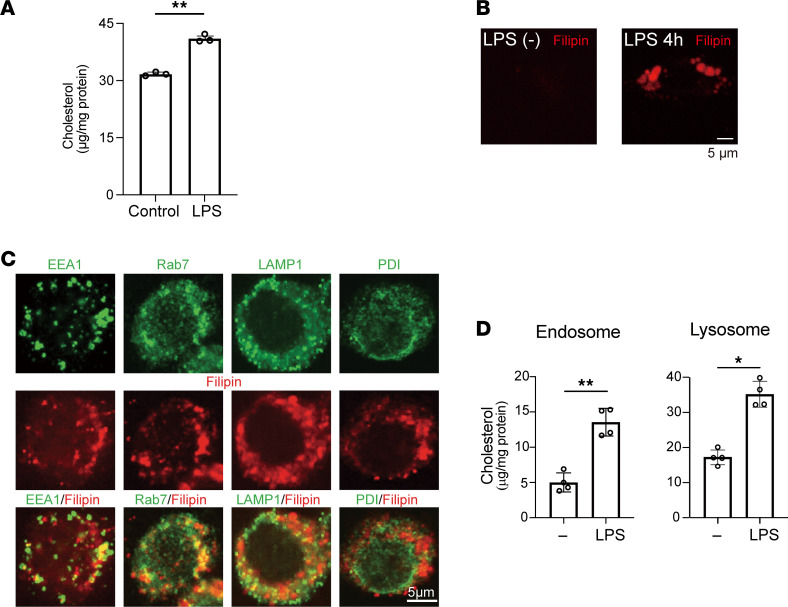
Cellular cholesterol accumulates in response to TLR4 activation in RAW cells. RAW cells cultured in medium containing 10% FBS were stimulated with or without LPS (100 ng/mL) for 4 hours. (**A**) Cellular unesterified free cholesterol was quantified using gas chromatography/mass spectrometry (GC/MS). *n* = 4 in each group. **P < 0.01, Student’s 2-tailed *t* test. (**B**) Cellular free cholesterol was stained with filipin. Scale bar, 5 μm. (**C**) Localization of filipin staining within early and late endosomes and lysosomes. RAW cells treated for 4 hours with LPS were stained with markers for organelles [EEA1 (early endosomes), Rab7 (late endosome), LAMP1 (lysosomes), and PDI (ER)] (green) and with filipin (red). Scale bar, 5 μm. (**D**) Cholesterol levels within endosomes and lysosomes were measured using GC/MS. *n* = 4 in each group. *P < 0.05, **P < 0.01, Student’s 2-tailed *t* test. Data shown as mean ± SD in all panels where *P* values are shown.

To begin investigating the potential integration of cellular cholesterol and inflammatory activation, we first assessed cholesterol levels in macrophages after inflammatory stimulation. RAW264.7 murine macrophages (hereafter referred to as “RAW cells”) were stimulated for 4 hours with LPS to activate TLR4 signaling, after which free (unesterified) cholesterol levels were analyzed using gas chromatography/mass spectroscopy (GC/MS). Levels of free cholesterol as well as intermediates in its biosynthetic pathway, including squalene, 2,3-oxidesqualene, lanosterol, lathosterol, and desmosterol, were all significantly increased by LPS stimulation (Figure 1A and Supplemental Figure 1; supplemental material available online with this article; https://doi.org/10.1172/jci.insight.138539DS1). The LPS-induced accumulation of free cholesterol was also visualized using filipin, which stains free cholesterol but not cholesteryl esters (Figure 1B) (28, 29). In contrast to LPS, 4 hours of stimulation with IL-4, which upregulated the M2 macrophage marker *Arg1* (Supplemental Figure 1C) (30), did not increase the cellular cholesterol or cholesterol synthesis intermediates (Supplemental Figure 1D).

Having observed that LPS greatly increases cellular cholesterol levels, we investigated the subcellular localization of cholesterol by staining RAW cells stimulated for 4 hours with LPS with filipin and selected organelle markers. The filipin signal colocalized in part with areas positive for EEA1 (early endosome marker), Rab7 (late endosomes), and LAMP1 (lysosomes) (Figure 1C and Supplemental Figure 2). By contrast, staining for the ER marker PDI was mostly separated from filipin-stained areas, as previously reported (15) (Supplemental Figure 2). Subcellular organelle isolation followed by cholesterol measurement using GC/MS also showed that free cholesterol levels were increased within endosomes and lysosomes in LPS-stimulated cells (Figure 1D).

Because plasma membrane lipid rafts are known to be involved in TLR4 signaling (23, 31), we stained RAW cells with FITC-labeled cholera toxin subunit B (CTB), which specifically binds GM1, a ganglioside enriched in membrane lipid rafts (Supplemental Figure 3). We detected no clear changes in GM1 staining in the plasma membrane, as previously suggested (32, 33). Although the semiquantitative nature of the staining means that further analyses will be required to clarify the dynamics of cholesterol and lipid rafts within the plasma membrane after TLR4 activation, the accumulation of intracellular cholesterol was much more pronounced than the changes in plasma membrane cholesterol.

### Cholesterol metabolism is activated by TLR4 activation.

**Figure 2:**
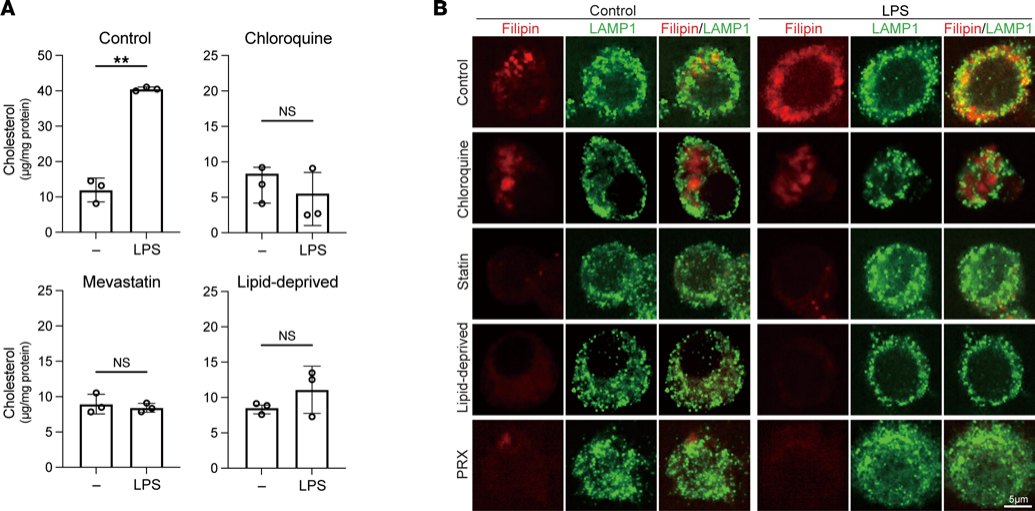
LPS activates multiple processes in cholesterol metabolism. (**A** and **B**) RAW cells were treated with mevastatin (50 μM) for 20 hours or chloroquine (100 μM) for 2 hours in medium containing FBS or were cultured in medium containing lipid-deprived FBS for 20 hours. The cells were then treated with LPS for 4 hours. In **A**, cholesterol levels in lysosomes were measured with GC/MS. *n* = 3 in each group. Mean ± SD. **P < 0.01, Student’s 2-tailed *t* test. In **B**, localization of the free cholesterol and lysosomes was visualized.

We next investigated the mechanisms underlying the increase of subcellular cholesterol. Cholesterol can be generated endogenously or acquired by cells via uptake of lipoproteins. Autophagy and lipophagy, a selective autophagy targeting lipid droplets, may also increase lysosomal cholesterol by taking cholesterol from membranes and lipid droplets. To assess the involvement of cholesterol synthesis and lipophagy, RAW cells were pretreated with mevastatin, an HMG-CoA reductase inhibitor, for 20 hours or chloroquine, an autophagy inhibitor (34), for 2 hours and then stimulated with LPS for 4 hours. Following the LPS stimulation, no intracellular cholesterol accumulation was observed in cells pretreated with mevastatin or chloroquine (Figure 2A). Filipin and LAMP1 staining showed that mevastatin blocked lysosomal accumulation of cholesterol (Figure 2B). Statins reportedly modulate multiple cellular processes involved in cholesterol metabolism, including cholesterol efflux (35), in addition to cholesterol synthesis. That said, increases in the levels of cholesterol biosynthesis intermediates (Supplemental Figure 1B) suggest activation of cholesterol synthesis contributes to the cellular cholesterol accumulation after LPS.

Though chloroquine did not change the localization of lysosomes visualized with LAMP1, filipin staining distributed in a droplet-like pattern within unstimulated RAW cells (Figure 2B). The pattern of filipin staining was not much affected by LPS and did not overlap the LAMP1 staining before or after LPS stimulation in chloroquine-treated cells. This suggests a relocation of cellular cholesterol from lysosomes. Because chloroquine inhibits autophagy by blocking the binding of autophagosomes to lysosomes through alteration of the lysosomal acidic environment, chloroquine might also affect lysosomal processes other than autophagy to relocate free cholesterol (34, 36).

To determine whether lipid uptake is also involved in the LPS-induced increase in subcellular cholesterol, RAW cells were cultured for 24 hours in medium containing 10% lipid-deprived FBS and then stimulated with LPS for 4 hours. The lack of FBS-derived lipids in the culture medium attenuated the LPS-induced cellular cholesterol accumulation (Figure 2, A and B). This result together with the others summarized in this section indicate that upregulation of cholesterol synthesis and cholesterol uptake both contribute to the increase in lysosomal cholesterol following LPS stimulation. Moreover, the results with chloroquine suggest the involvement of autophagy/lipophagy. Our data thus demonstrate that LPS activates multiple processes involved in cholesterol metabolism and that accumulation of cholesterol within endosomes and lysosomes (endolysosomes) mark those metabolic changes.

### Cholesterol accumulation is required for inflammatory activation of macrophages.

**Figure 3:**
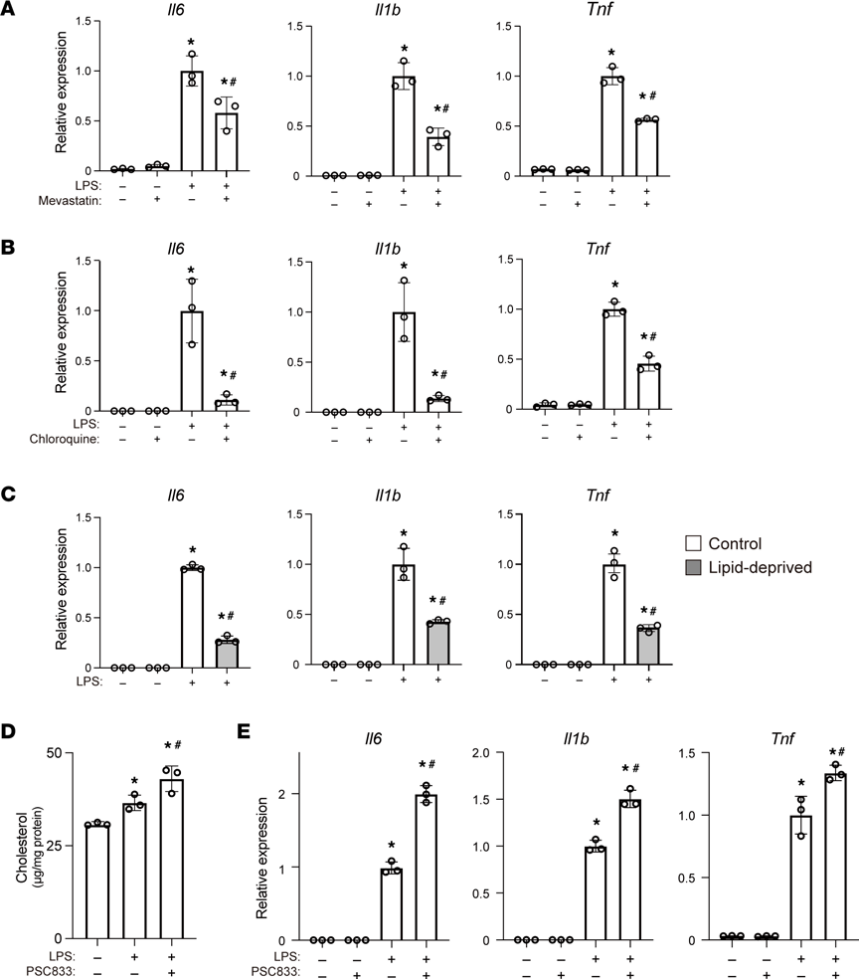
Accumulation of free cholesterol within lysosomes is necessary for inflammatory activation of RAW cells. (**A** and **B**) RAW cells were treated with or without mevastatin (**A**) or chloroquine (**B**) for 20 or 2 hours, respectively, prior to 4 hours of LPS stimulation. Relative expression levels of *Il6*, *Il1b*, and *Tnf* mRNA were determined using quantitative PCR (qPCR). *n* = 3 per condition, *P < 0.05 vs. LPS-untreated cells, ^#^*P* < 0.05 vs. control LPS only–treated cells, Tukey-Kramer post hoc test. (**C**) Expression of *Il6*, *Il1b*, and *Tnf* mRNA in RAW cells cultured in lipid-deprived media with or without LPS stimulation. *P < 0.05 vs. LPS-untreated cells, ^#^*P* < 0.05 vs. control LPS-treated cells cultured in control media, Tukey-Kramer post hoc test. (**D**) RAW cells were treated with PSC-833 (40 μM) for 20 hours followed by LPS for 4 hours. Cellular cholesterol was quantified using GC/MS. *n* = 3 in each group. *P < 0.05 vs. LPS-untreated cells, ^#^*P* < 0.05 vs. LPS only–treated cells, Tukey-Kramer post hoc test. (**E**) Expression of *Il6*, *Il1b*, and *Tnf* mRNA in RAW cells treated with or without PSC-833 for 20 hours followed by LPS for 4 hours. *n* = 3 in each group. *P < 0.05 vs. LPS‑untreated cells, ^#^*P* < 0.05 vs. PSC-833-untreated cells, Tukey-Kramer post hoc test. Mean ± SD (all panels).

Having detected cholesterol accumulation within lysosomal/endosomal regions after LPS stimulation, we tested the idea that cellular cholesterol accumulation is causally required for macrophage activation by assessing proinflammatory gene expression in RAW cells treated with mevastatin or chloroquine followed by LPS. Induction of the proinflammatory cytokine genes *Il6*, *Il1b*, and *Tnf* was significantly suppressed in the mevastatin-treated cells, as previously reported (37) (Figure 3A, Supplemental Table 1). Chloroquine similarly inhibited induction of these cytokine genes (Figure 3B). In addition, lipid removal from the culture medium also significantly decreased the induction of *Il6*, *Il1b*, and *Tnf* mRNAs (Figure 3C). These results support the notion that activation of cholesterol metabolism is important for the regulation of LPS-induced proinflammatory gene expression in macrophages.

We next asked whether inhibition of cholesterol efflux also affects cellular cholesterol and inflammatory responses in LPS-stimulated RAW cells. Excess free cholesterol is removed from macrophages through active transfer mediated by cholesterol transporters as well as passive transmembrane diffusion (20). Cholesterol transporters, including SR-BI, ABCA1, and ABCG1, all contribute to cholesterol efflux from macrophages (20, 21, 23, 38). We first tested whether cellular cholesterol levels are increased by the ABCA1 inhibitor PSC-833 (39). As expected, PSC-833 further increased cellular cholesterol levels in LPS-treated cells (Figure 3D) and led to superactivation of the 3 tested proinflammatory cytokine genes (Figure 3E). siRNA-mediated *Abca1* knockdown likewise led to cellular cholesterol accumulation (Supplemental Figure 4, A and B) and enhanced proinflammatory cytokine gene expression (Supplemental Figure 4C), as was reported previously (22). On the other hand, *Abcg1* knockdown did not increase cellular cholesterol levels, nor did it affect proinflammatory cytokine gene expression (Supplemental Figure 4, B and C). Moreover, simultaneously suppressing *Abca1* and *Abcg1* did not produce an additive effect on cytokine gene expression (Supplemental Figure 4C). These results show that increasing accumulation of cellular cholesterol by inhibiting its efflux enhances the inflammatory response and that ABCA1 is a major mediator of cholesterol efflux from LPS-treated macrophages.

### Cellular cholesterol is crucial for Myd88 signaling.

**Figure 4:**
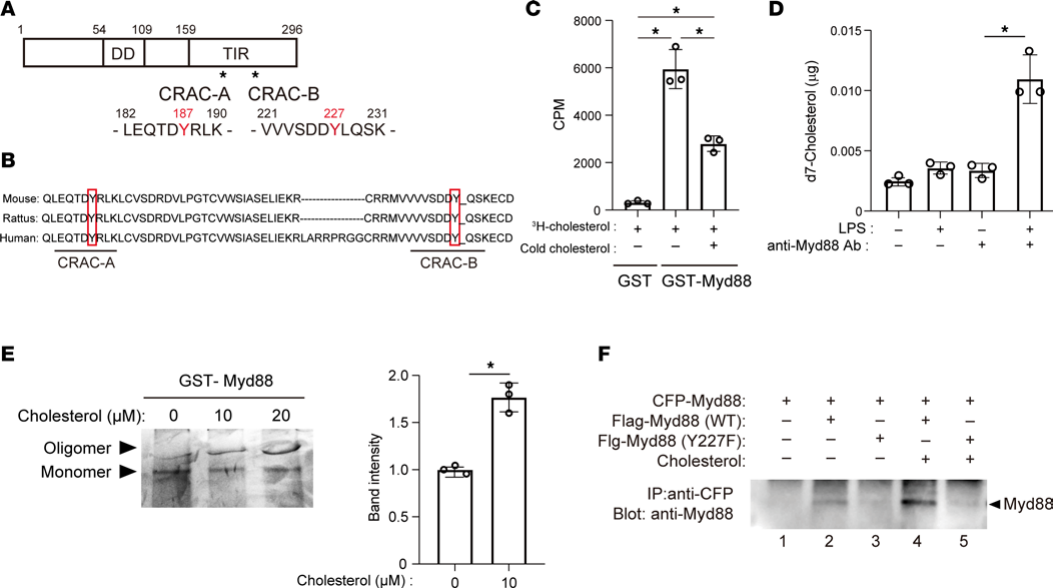
Cholesterol binds to the Myd88 CRAC domain. (**A**) Schematic representation of mouse Myd88 protein. There are 2 CRAC sequences in its C-terminal Toll/interleukin 1 receptor (TIR) domain. DD, death domain. (**B**) Myd88 amino acid sequences around 2 CRAC sequences in human, rat, and mouse. (**C**) Affinity-purified, recombinant, GST-tagged Myd88 or GST was incubated with ^3^H‑cholesterol (10 μM). For the competition experiment, unlabeled cholesterol (10 μM) was added. GST-Myd88 and GST were recovered, and radioactivity was measured. *P < 0.05. Tukey-Kramer post hoc test. (**D**) RAW cells were cultured for 24 hours in medium containing deuterium-labeled cholesterol (d7-cholesterol) (10 μg/mL), with or without 4 hours of LPS stimulation. Whole-cell lysates were then extracted, and the amount of d7-cholesterol pulled down by Myd88 antibody was detected with GC/MS. *P < 0.05 vs. LPS-untreated cell lysates pulled down with anti-Myd88 antibody. (**E**) Purified GST-Myd88 was incubated with cholesterol, run on a Native polyacrylamide gel, and visualized by silver staining. Relative band intensities corresponding to oligomeric Myd88 are shown in the bar graph. *n* = 3 in each group. *P < 0.05. Student’s 2-tailed *t* test. (**F**) BMDMs were transfected with plasmids expressing Flag-tagged WT or Y227F mutant Myd88 along with CFP-tagged Myd88 and treated with or without cholesterol (10 μM) for 4 hours. Whole-cell lysates were collected and subjected to immunoprecipitation analysis. Data shown are representative of 3 independent experiments. Data shown as mean ± SD in all panels where *P* values are shown.

Our findings so far suggest that the regulation of cellular cholesterol is integral to the inflammatory response of macrophages to TLR4 signaling. To determine how activation of cholesterol metabolism is linked to inflammatory gene expression, we sought the mechanism by which accumulated cholesterol influences TLR4 signaling. Myd88 is the canonical adaptor protein for the inflammatory signaling pathways downstream of most TLRs, including TLR4 (40). We found 2 cholesterol recognition amino acid consensus (CRAC) sequences (L/V-X (1-5)-Y-X (1-5)-R/K) within the murine Myd88 protein. The central tyrosine (Y) residues are at positions 187 (CRAC-A) and 227 (CRAC-B) (Figure 4A), and the CRAC sequences containing those tyrosines are well conserved in mouse, rat, and human Myd88 (Figure 4B).

The presence of CRAC sequences prompted us to assess the binding of cholesterol to Myd88. We found that recombinant GST-tagged Myd88 associated with ^3^H-labeled cholesterol in vitro (Figure 4C). Addition of unlabeled cholesterol competitively decreased the radioactivity of GST-Myd88, which is indicative of the specific binding of cholesterol to Myd88. NBD-cholesterol fluorescence binding assays (41) showed that cholesterol binds to the Myd88 CRAC domain with a *K_D_* of 14.1 μM.

To determine whether Myd88 protein binds cholesterol in live cells, RAW cells were cultured for 20 hours in medium containing deuterium-labeled cholesterol (d7-cholesterol), followed by 4 hours of LPS stimulation. The amount of d7-cholesterol pulled down with Myd88 was detected with GC/MS. The results showed that d7-cholesterol was significantly increased in Myd88-containing complexes from LPS-activated cells as compared with untreated cells (Figure 4D). This indicates that d7-cholesterol binds to endogenous Myd88 in response to LPS.

Higher order self-assembly of oligomeric Myd88 is essential for amplification of TLR signaling (42). We therefore tested whether cholesterol binding is important for the self-oligomerization of Myd88 proteins. GST-tagged mouse Myd88 was incubated with or without cholesterol and then run on a native polyacrylamide gel. Addition of cholesterol increased the intensity of the higher molecular weight band corresponding to oligomerized Myd88 (Figure 4E). To confirm the cholesterol requirement for Myd88 oligomerization, mouse bone marrow–derived macrophages (BMDMs) were transfected with plasmids encoding Flag-tagged WT or Y227F mutant Myd88 along with CFP-tagged Myd88 and cultured with or without cholesterol. The cell lysates were then immunoprecipitated with anti-CFP antibody (Figure 4F). WT Flag-Myd88 was pulled down with anti-Flag antibody, and the intensity of the WT-Myd88 band was higher in lysates from cells treated with cholesterol (Figure 4F, lane 2 vs. 4). A mutation within the CRAC-B domain (Y227F) in Flag-Myd88 nearly eliminated coimmunoprecipitated Flag-Myd88 (Figure 4F, lanes 3 and 5), demonstrating that the CRAC-B domain is essential for binding between 2 Myd88 proteins.

**Figure 5:**
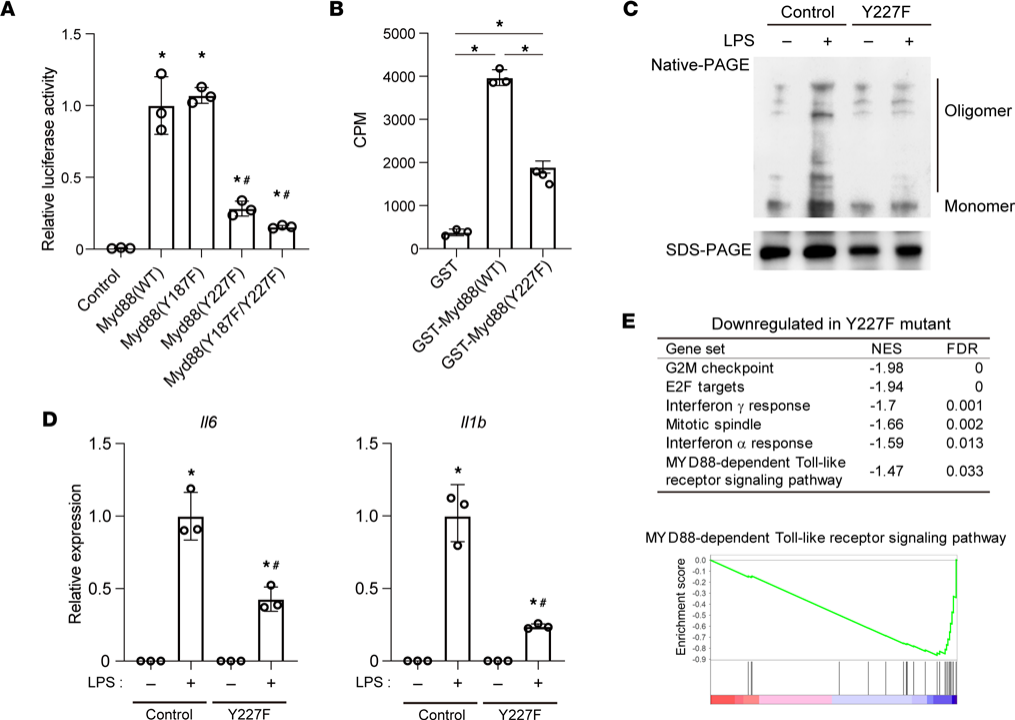
Cholesterol binding to the Myd88 CRAC domain is required for signal amplification. (**A**) HEK293T cells were transfected with plasmids encoding WT Myd88 or the indicated Myd88 mutant and a NF-κB motif-driven luciferase reporter plasmid (NFκB-Luc). Luciferase activity was normalized to that of cells transfected with a WT Myd88 expression vector. *n* = 3/group. *P < 0.05 vs. cells transfected with control plasmid. ^#^*P* < 0.05 vs. cells transfected with plasmids encoding WT Myd88. Tukey-Kramer post hoc test. Data are representative of 3 independent experiments. (**B**) Affinity-purified, recombinant GST-tagged Myd88 (WT), Myd88 (Y227F), or GST was incubated with ^3^H-cholesterol (10 μM) as shown in Figure 4B. GST-Myd88 or GST was recovered, and radioactivity was measured. *n* = 3. *P < 0.05. Tukey-Kramer post hoc test. (**C**) Whole-cell lysates were collected from control and Myd88-Y227F mutant RAW cells, stimulated for 4 hours with or without LPS, subjected to Native PAGE, and blotted with an anti-Myd88 antibody. Bands with a slower migration rate correspond to the oligomerized form of Myd88 proteins. A photograph of the same protein run on an SDS-PAGE gel and blotted with anti-Myd88 antibody is shown as a control at the bottom. Data shown are representative of 3 independent experiments. (**D**) Control and Myd88-Y227F mutant RAW cells were stimulated for 4 hours with LPS, after which expression of *Il6* and *Il1b* was analyzed with qPCR. *n* = 3/group. *P < 0.05 vs. LPS-untreated control cells, ^#^*P* < 0.05 vs. LPS-treated control cells. Tukey-Kramer post hoc test. (**E**) GSEA of MSigDB hallmark gene sets and GO Myd88-dependent TLR signaling pathways. RNA-Seq results for control and Y227F mutant cells stimulated with LPS for 4 hours were used. Shown are the gene sets downregulated (FDR < 0.05) in the Y227F cells. An enrichment plot of Myd88 pathway is also shown. No gene sets were upregulated (FDR < 0.05). Data shown as mean ± SD in all panels where *P* values are shown.

The functional impact of cholesterol binding to Myd88 was then assessed by overexpressing Myd88 mutants in which phenylalanine was substituted for tyrosine within the CRAC domains (Y187F and/or Y227F) in HEK293T cells. Downstream NF‑κB activation was monitored using a luciferase reporter under the control of NF-κB binding motifs (NFκB-Luc) (43). Overexpression of WT Myd88 greatly increased luciferase activity (Figure 5A). This effect was strongly suppressed by the Y227F mutation but not by the Y187F mutation, which indicates that the CRAC-B domain containing Y227 is essential for Myd88 function. In line with that, ^3^H‑cholesterol binding to recombinant GST-Myd88 was significantly impaired by the Y227F mutation (Figure 5B). These results strongly suggest that cholesterol binds to the CRAC-B domain of Myd88 and promotes the protein’s self-oligomerization, thereby amplifying downstream signaling.

To further confirm the necessity of the Y227 residue of Myd88 for LPS‑dependent macrophage oligomerization, RAW cells harboring a Myd88 Y227F mutant were established using CRISPR/Cas9 technology (Supplemental Figure 5). Myd88 Y227F mutant and control cells were then stimulated for 4 hours with LPS, after which whole-cell lysates were analyzed with native PAGE. Levels of oligomerized Myd88 detected as slowly migrating forms were increased after LPS stimulation in the control cells but not the Y227F mutant cells (Figure 5C), which demonstrates that Y227 is essential for LPS-induced oligomerization of Myd88.

In addition, LPS-mediated induction of *Il6* and *Il1b* was significantly suppressed in the Y227F mutant cells (Figure 5D). Gene set enrichment analysis (GSEA) of Molecular Signatures Database (MSigDB) hallmark gene sets and gene ontology (GO) analysis of the Myd88-dependent TLR signaling pathway showed that gene sets related to inflammation and Myd88 signaling were downregulated (FDR < 0.05) in the Y227F mutant cells as compared with control cells after 4 hours of LPS treatment (Figure 5E). Collectively, these data indicate that cholesterol binding to the CRAC-B domain of Myd88 is crucial for innate immune signaling via the TLR4/Myd88 pathway.

### Polyrotaxane promotes cholesterol efflux and reduces cellular cholesterol.

The results summarized so far demonstrate that the dynamic regulation of cholesterol metabolism that leads to increased cellular cholesterol is integral to LPS‑induced proinflammatory activation of macrophages. They also indicate that endolysosomal cholesterol accumulation is a hallmark of the LPS response and that interfering with cholesterol metabolic processes to decrease endolysosomal cholesterol suppresses inflammatory activation (Figure 3, A–C). Based on those findings, we hypothesized that pharmacologically reducing endolysosomal cholesterol may be an effective means of regulating the inflammatory response of macrophages. The supramolecular compound polyrotaxane (PRX) is composed of multiple β-cyclodextrins (βCDs) threaded along a polymer chain capped with acid-cleavable bulky stopper molecules (44, 45). After PRX is taken into cells, the acidic environment within endolysosomes releases the βCD molecules from the polymer chain (44). As expected, fluorescently labeled PRX preferentially accumulated within late endosomes and lysosomes in RAW cells (44, 46) (Supplemental Figure 6A).

βCD molecules, which contain a hydrophobic cavity, have been shown to extract cholesterol from plasma membranes when added to culture medium and to disrupt lipid rafts, which are rich in cholesterol and sphingolipids (47). By contrast, PRX only releases βCD after transfer to lysosomes, making it unlikely that the freed βCD molecules directly attack plasma membrane cholesterols (44). To support that, we treated RAW cells with PRX containing 4 mM βCD or with 4 mM 2-hydroxypropyl-βCD. The microdomain/lipid raft structure was preserved in PRX-treated cells, as demonstrated by the binding of FITC‑labeled CTB, which binds to the microdomain-associated ganglioside GM1. By contrast, 2-hydroxypropyl-βCD disrupted the raft structure (Supplemental Figure 6B).

**Figure 6:**
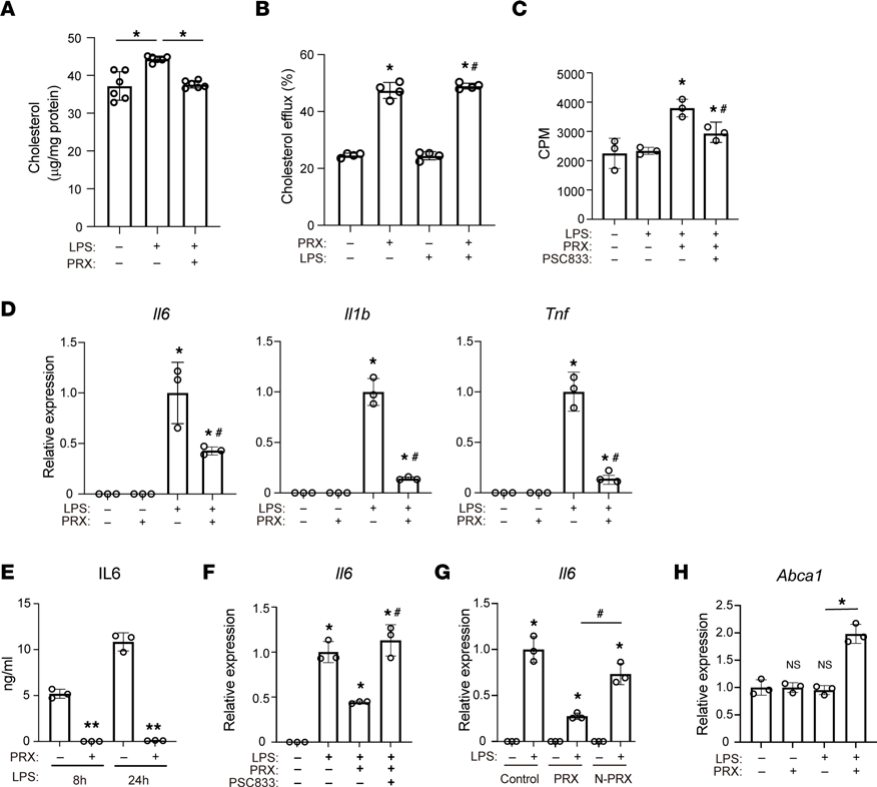
PRX decreases endolysosomal cholesterol. (**A**) RAW cells were first treated with or without PRX (2 mM βCD) for 20 hours then with LPS for 4 hours. Cellular cholesterol was quantified with GC/MS (*n* = 6). *P < 0.05. Tukey-Kramer post hoc test. (**B**) Effects of PRX on cholesterol efflux. *n* = 4 in each group. *P < 0.05 vs. untreated cells, ^#^*P* < 0.05 vs. LPS only–treated cells. Tukey-Kramer post hoc test. (**C**) Effects of PSC-833 on cholesterol efflux. *n* = 4. *P < 0.05 vs. untreated cells, ^#^*P* < 0.05 vs. PRX plus LPS-treated cells, Tukey-Kramer post hoc test. (**D**) Expression of *Il6*, *Il1b*, and *Tnf* mRNA in RAW cells treated with PRX for 20 hours, followed by LPS stimulation for 4 hours. *P < 0.05 vs. LPS-untreated cells, ^#^*P* < 0.05 vs. LPS only–treated cells, Tukey-Kramer post hoc test. (**E**) RAW cells were treated with or without PRX and LPS for the indicated times. IL-6 protein in the conditioned medium was quantified using an ELISA. *n* = 3 in each group. Data shown are representative of 3 independent experiments. **P < 0.01 vs. PRX-untreated cells at each time point. Student’s 2-tailed *t* test. (**F**) RAW cells were treated for 20 hours with PRX and/or PSC-833 prior to 4 hours with LPS. Expression of *Il6* mRNA was assessed with qPCR. *P < 0.05 vs. LPS-untreated cells, ^#^*P* < 0.05 vs. PRX and LPS-treated cells. (**G**) RAW cells were treated for 20 hours with PRX or N-PRX prior to 4 hours with LPS. Expression of *Il6* mRNA was assessed with qPCR. *P < 0.05 vs. LPS-untreated cells, ^#^*P* < 0.05 vs. PRX and LPS-treated cells, Tukey-Kramer post hoc test. (**H**) Expression of *Abca1* mRNA in RAW cells treated with PRX for 20 hours, followed by stimulation with LPS for 4 hours. *P < 0.05 vs. control cells. Tukey-Kramer post hoc test. Data shown as mean ± SD in all panels where *P* values are shown.

PRX releases βCD molecules within endolysosomes, where βCD may bind cholesterol and promote its trafficking and efflux. To test that idea, we used GC/MS to measure cellular free cholesterol and found that PRX reduced LPS-induced accumulation of cellular cholesterol (Figure 6A). Lysosomal cholesterol accumulation after LPS was also attenuated by PRX (Figure 2B). Moreover, cholesterol efflux assays (48) showed that PRX greatly increased cholesterol efflux, irrespective of LPS treatment (Figure 6B). Inhibition of ABCA1 with PSC-833 partially inhibited PRX‑activated cholesterol efflux (Figure 6C). These findings indicate that PRX reduces endolysosomal cholesterol in part by enhancing cholesterol efflux via the ABCA1 transporter.

### PRX suppresses inflammatory activation in macrophages.

Subsequent investigation of the effects of PRX on inflammatory responses in RAW cells revealed that it significantly decreased LPS-mediated induction of *Il6*, *I1b*, and *Tnf* (Figure 6D). PRX also significantly inhibited *IL6* induction elicited by LPS (Figure 6E). Inhibition of cholesterol efflux via the ABCA1 transporter with PSC-833 canceled the reduction of *Il6* elicited by PRX (Figure 6F).

To verify that release of βCDs from PRX within endolysosomes is required for the antiinflammatory effect of PRX, we made use of a PRX form that could not be disassembled (N-PRX). N-PRX is threaded along a polymer chain capped with acid‑stable bulky stopper molecules so that βCDs are not released within endolysosomes. N-PRX failed to suppress LPS-induced *Il6* expression (Figure 6G), suggesting βCDs released from PRX within lysosomes suppress inflammatory activation of RAW cells.

### PRX inhibits TLR4/Myd88 signaling.

**Figure 7:**
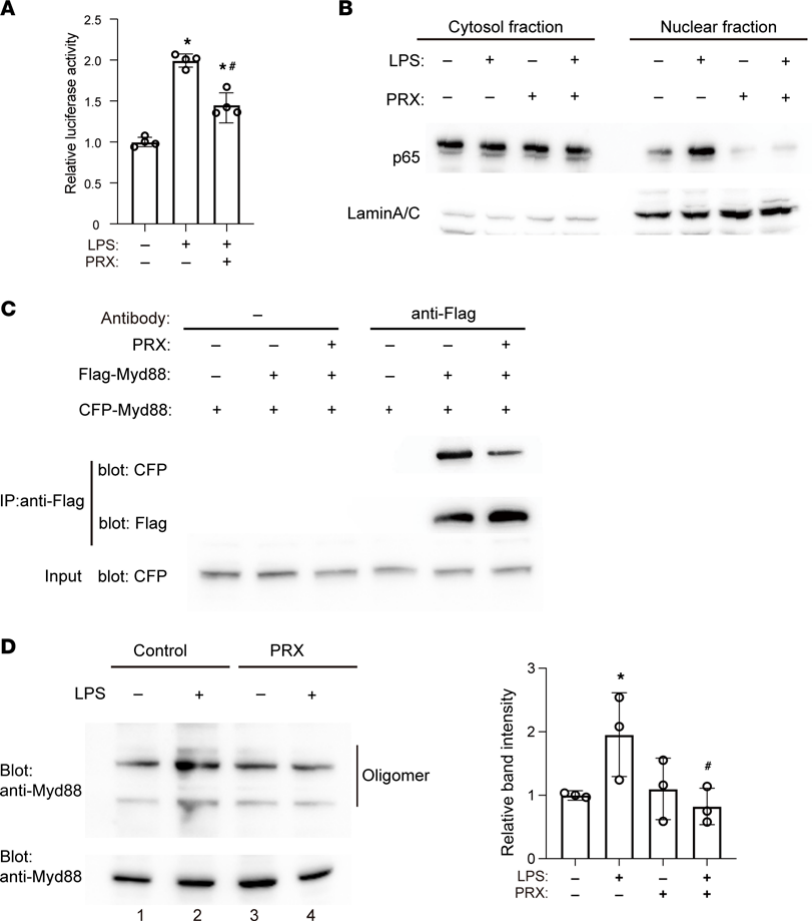
PRX inhibits the TLR4/Myd88/NF-κB pathway. (**A**) RAW cells transfected with NFκB-Luc were cultured in the presence or absence of PRX for 20 hours and treated with LPS or vehicle for 4 hours. Luciferase activities are shown relative to the activity in cells transfected with NFκB-Luc with no treatment. *P < 0.05 vs. LPS-untreated cells, ^#^*P* < 0.05 vs. PRX-untreated cells, Tukey-Kramer post hoc test. (**B**) RAW cells were treated for 20 hours with or without PRX and then for 4 hours with or without LPS. Nuclear and cytoplasmic fractions were prepared from cell lysates and subjected to Western blotting for the NF-κB p65 subunit. Lamin A/C was used as an internal control for nuclear protein. Representative blots from 3 individual experiments are shown. (**C**) PRX decreased interaction between CFP-Myd88 and Flag-Myd88. HEK293T cells were transfected with CFP-Myd88 expression plasmids with or without Flag-Myd88. The cell lysates were subjected to immunoprecipitation using anti-Flag antibody. Representative blots from 3 individual experiments are shown. (**D**) Whole-cell lysates from RAW cells were subjected to native PAGE. Bands with slow migration correspond to the oligomerized forms of Myd88 protein, as indicated. A photograph of the same protein run on an SDS-PAGE gel is shown as a control at the bottom. Relative band intensity corresponding to the oligomerized Myd88 compared with that of LPS-untreated control cells are shown in the bar graph. *n* = 3 in each group. *P < 0.05 vs. unstimulated cells, ^#^*P* < 0.05 vs. LPS only–treated cells. Tukey-Kramer post hoc test. Data shown as mean ± SD in all panels where *P* values are shown.

Having shown that PRX inhibits inflammatory gene expression triggered by TLR4 activation, we further tested the effect of PRX on TLR4/Myd88/NF‑κB signaling. PRX diminished LPS-induced activation of a NF-κB reporter (Figure 7A) and inhibited LPS‑induced nuclear translocation of the NF-κB p65 subunit (Figure 7B and Supplemental Figure 6C) in RAW cells. This suggests PRX suppresses NF-κB activation via TLR4/Myd88 signaling.

We next tested the effect of PRX on Myd88 oligomerization by expressing Flag‑tagged and CFP-tagged Myd88 proteins in HEK293T cells and then immunoprecipitating Flag-Myd88–containing complexes (Figure 7C). Whereas CFP‑Myd88 was pulled down with Flag-Myd88 in lysates from control cells, the intensity of the CFP-Myd88 band was decreased in lysates from PRX-treated cells. This prompted us to test whether PRX suppresses endogenous Myd88 oligomerization in RAW cells, which revealed that PRX suppresses LPS-induced increases in oligomerized Myd88, as indicated by the decreased intensity of the oligomerized Myd88 band (Figure 7D, lane 1 vs. 2 and lane 3 vs. 4). Given that cholesterol promotes Myd88 oligomerization, which is essential for Myd88 signal amplification (Figure 4, E and F), these results suggest that PRX most likely inhibits Myd88 oligomerization by reducing the availability of cholesterol for Myd88.

Because Myd88 is a canonical adaptor protein for most TLRs (not TLR3), we also assessed the effects of PRX on other pattern recognition receptors by treating RAW cells with agonists for TLR9 (CpGB), TLR3 (polyI-C), and RNA sensor RIG-I (3pRNA). Like TLR4, TLR9 signals through Myd88, but TLR3 and RIG‑I do not. Whereas CpGB-mediated induction of *Il6* was diminished by PRX, induction of *Il6* mediated by 3pRNA or polyI-C was unaffected or even enhanced by PRX (Supplemental Figure 6D). This suggests that although PRX inhibits Myd88-dependent TLR4 and TLR9 signaling, it does not inhibit Myd88‑independent TLR3 and RIG-I signaling.

### PRX suppresses inflammatory responses in BMDMs.

**Figure 8:**
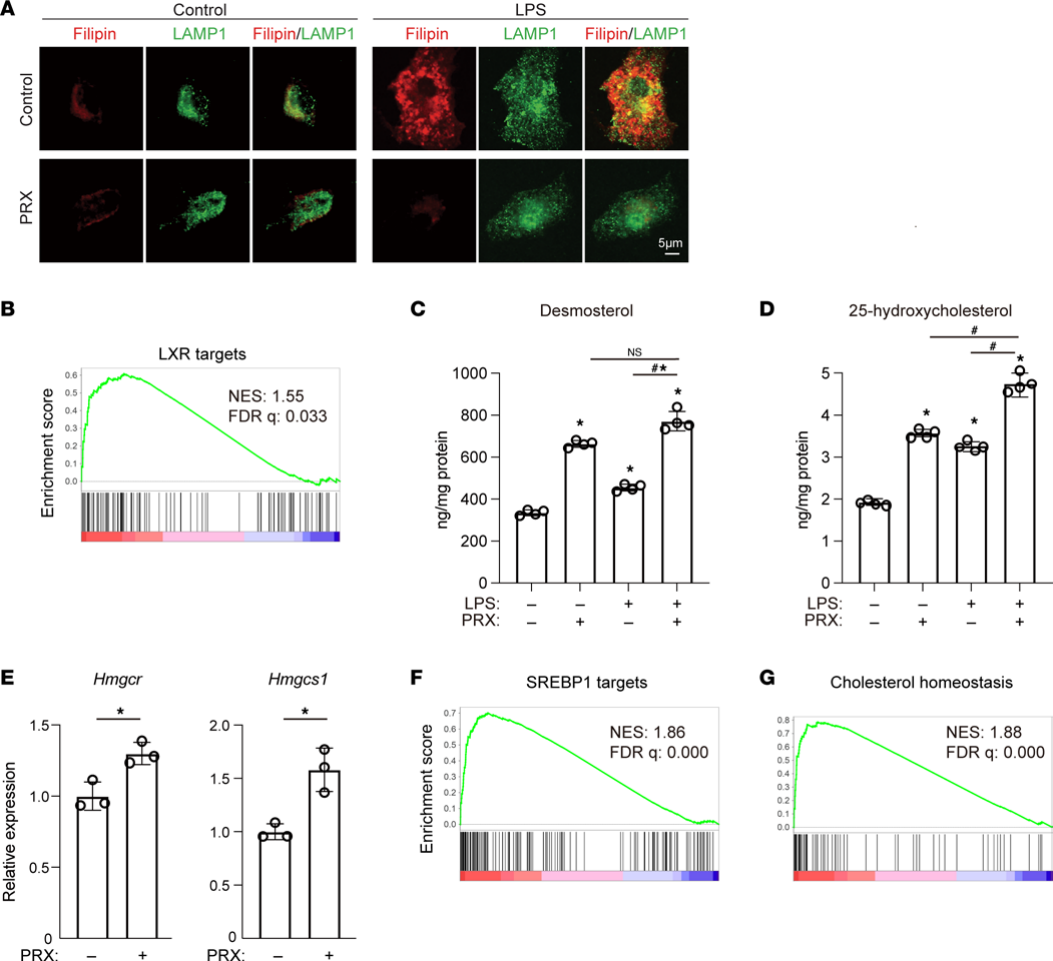
PRX suppresses inflammatory responses and modulates cholesterol metabolism in BMDMs. (**A**) BMDMs were treated with or without PRX (2 mM βCD) for 20 hours followed by LPS or vehicle for 4 hours. Filipin and LAMP1 staining are shown. Scale bar, 5 μm. (**B**) GSEA enrichment score for LXR target genes in PRX-treated BMDMs as compared with untreated BMDMs. (**C** and **D**) RAW cells were treated for 20 hours with or without PRX (2 mM βCD) prior to 4 hours with LPS. Cellular levels of desmosterol (**C**) and 25-hydroxycholesterol (**D**) were assessed by GC/MS. *P < 0.05 vs. untreated cells, ^#^*P* < 0.05, Tukey-Kramer post hoc test. (**E**) Expression of *Hmgcr* and *Hmgcs1* mRNA in RAW cells. *P < 0.05, Student’s 2-tailed *t* test. Data shown as mean ± SD in all panels where *P* values are shown. (**F** and **G**) GSEA enrichment scores in PRX-treated BMDMs as compared with untreated BMDMs. The SREBP1 target gene set consisting of the genes with SREBP1 peaks in their regulatory regions (**F**) and that for hallmark cholesterol homeostasis (**G**) were analyzed.

We also tested whether primary BMDMs exhibit similar cholesterol metabolic responses to LPS. Using immunofluorescence imaging, we found that 4 hours of LPS stimulation increased the filipin signal, which at least partially colocalized with the LAMP1-positive area in BMDMs (Figure 8A). As in RAW cells, cholesterol accumulation was diminished by pretreatment with PRX (Figure 8A).

We then used RNA-Seq to investigate the gene sets affected by PRX in LPS‑activated BMDMs. We found that among 9,479 RefSeq genes with regularized log transformed counts greater than 7 in at least 1 sample at any time point in the vehicle-treated samples, the expression levels of 2,026 genes were significantly (FDR < 0.05) increased by ≥2-fold after 4 hours of LPS treatment (Supplemental Figure 7A). These LPS-upregulated genes were highly enriched among genes belonging to gene sets related to defense response and cytokine production (Table 1). This LPS-induced upregulation was suppressed by PRX, which significantly (FDR < 0.05) downregulated 376 genes enriched in genes belonging to gene sets related to inflammatory response and cytokine signaling (Table 1). The antiinflammatory effect of PRX was observed over the course of the inflammatory response, as shown in a heatmap of LPS-induced genes; however, βCD did not have similar effects on the genes (Supplemental Figure 7A). qPCR analysis independently verified that PRX significantly suppressed expression of inflammatory genes, including *Il6*, *Il1b*, *Il12b*, *Cxcl9*, *Cxcl1*, and *Ccl9*, in BMDMs after 4 hours of LPS stimulation (Supplemental Figure 7B). These findings indicate that PRX inhibits inflammatory activation of BMDMs by LPS.

### PRX enhances cholesterol efflux by activating liver X receptor.

As mentioned, PRX enhances cholesterol efflux from macrophages in part via the ABCA1 transporter (Figure 6, A–C). Notably, PRX increased *Abca1* mRNA expression in LPS-stimulated RAW cells (Figure 6H), suggesting PRX-induced upregulation of *Abca1* expression is an important action contributing to the enhanced cholesterol efflux. Because *Abca1* is a known target of liver X receptors (LXRs), we hypothesized that PRX affects expression of LXR target genes. To test that idea, we curated potential LXR targets using publicly available RNA-Seq and ChIP‑Seq data. Using the RNA-Seq data (Gene Expression Omnibus GSE90615), we identified genes that were significantly upregulated by the LXR agonist GW3965. We also used GREAT (49) to identify possible direct targets of LXR by detecting genes situated near LXR-α binding sites determined with ChIP-Seq. We then constructed a gene set consisting of the intersection between GW3965-activated genes and LXR-α binding targets. GSEA of RNA-Seq data from BMDMs stimulated with or without PRX followed by stimulation for 4 hours with LPS showed that LXR target genes were upregulated in the cells treated with PRX (Figure 8B).

Desmosterol and 25-hydroxycholesterol are reported to be potent activators of LXR in macrophages (50). The cellular levels of both were significantly increased with LPS or PRX stimulation alone but were further increased in the cells treated with PRX followed by LPS (Figure 8, C and D). This suggests PRX activates LXR by increasing levels of desmosterol and 25-hydroxycholesterol. LXR may in turn affect cholesterol metabolism and inflammatory gene expression, in part by enhancing cholesterol efflux via ABCA1 (51). In addition to its effect on LXR targets, PRX increased expression of the known SREBP target genes *Hmgcr* and *Hmgcs1* (Figure 8E) and the SREBP1 target gene set (Figure 8F). Moreover, PRX upregulated the gene set involved in the maintenance of cholesterol homeostasis (Figure 8G), which implies that PRX enhances cholesterol synthesis, thereby increasing desmosterol production. These findings indicate PRX regulates cellular cholesterol metabolism in macrophages via LXR and SREBP1.

### PRX attenuates atherogenesis in Ldlr^–/–^ mice.

The observation that Myd88-dependent inflammatory activation was blunted by PRX led us to test whether PRX could also modulate atherogenesis in vivo. LDL receptor–knockout (*Ldlr*^–/–^) mice, a widely used hypercholesterolemic atherosclerosis model, were fed a high-cholesterol diet with or without subcutaneous PRX administration (1,000 mg/kg body weight, every 2 days). This PRX treatment did not affect body weight or serum cholesterol profiles over the treatment period (Supplemental Figure 8).

**Figure 9:**
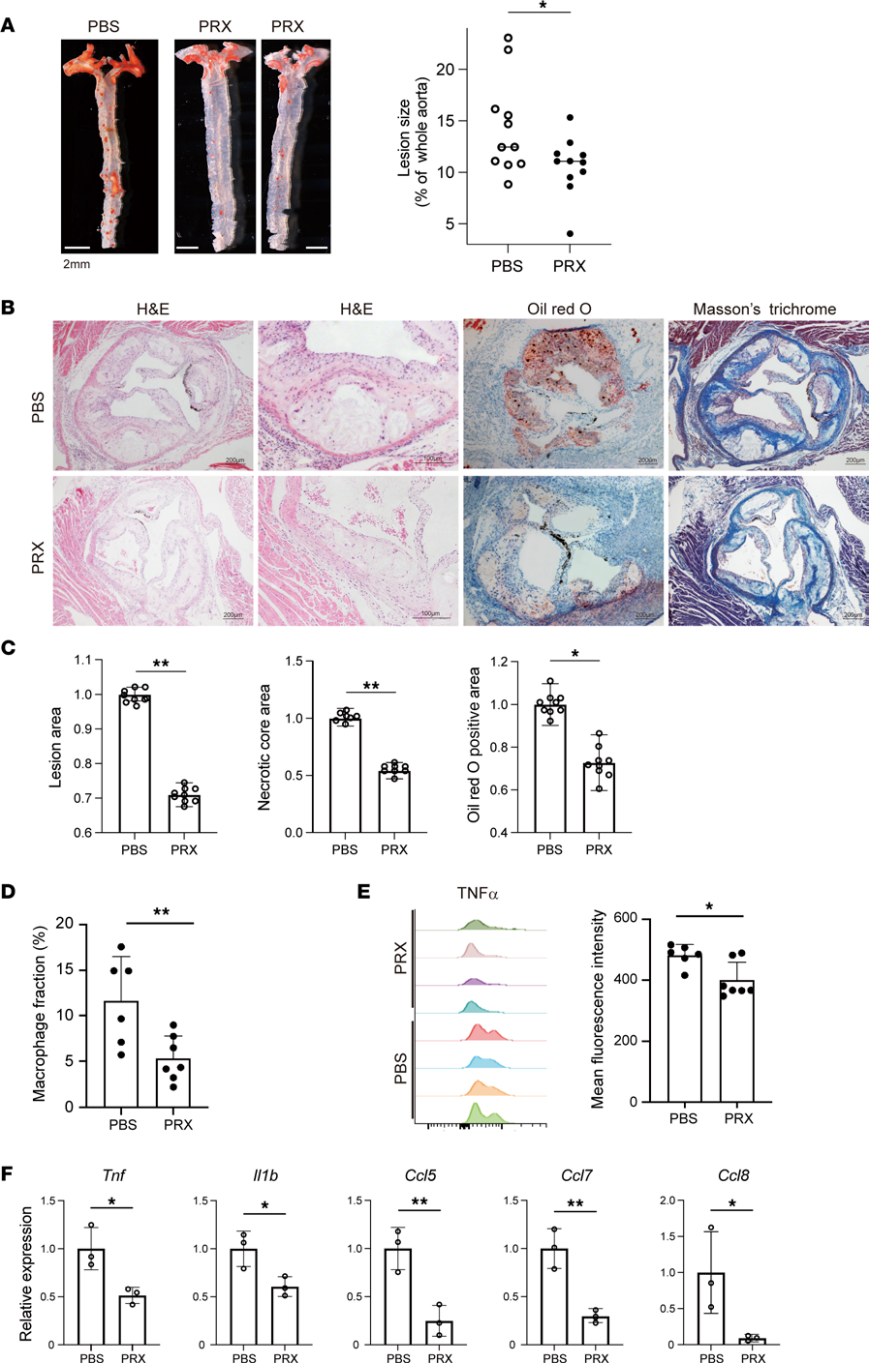
PRX suppresses atherogenesis in *Ldlr*^–/–^ mice. (**A**) Male *Ldlr*^–/–^ mice were fed a high-cholesterol diet for 11 weeks, with or without subcutaneous PRX injection (1,000 mg/kg BW every 2 days). Aortic atheromatous lesions were visualized after staining with Oil Red O. Oil Red O–positive areas were quantified and normalized with the entire aortic area. *n* = 11 in each group. *P < 0.05. Student’s 2-tailed *t* test. (**B**) Representative photographs of aortic sinuses from *Ldlr*^–/–^ mice treated with PBS or PRX. Sections were analyzed by staining with hematoxylin-eosin (H&E), Oil Red O, and Masson’s trichrome. Scale bars, 200 μm or 100 μm (second H&E column). (**C**) Atherosclerotic lesion, necrotic core, and Oil Red O–positive areas were quantified and normalized to the control values (PBS). *n* = 10 in each group. **P < 0.01, *P < 0.05. Student’s 2-tailed *t* test. (**D**) Flow cytometric analysis of cells from thoracic aortas of mice treated with PRX or PBS. Shown are fractions of CD11b^+^Ly6G^–^F4/80^+^ macrophages among the total live cells. *n* = 6–7 mice for each group. **P < 0.01, Student’s 2-tailed *t* test. (**E**) TNF-α expression in CD11b^+^Ly6G^–^F4/80^+^ macrophages. Note that there were fewer cells expressing higher levels of TNF-α in the PRX-treated group. *n* = 4 mice for each group. *P < 0.05, Student’s 2-tailed *t* test. (**F**) Relative mRNA expression of proinflammatory genes in the aorta. *P < 0.05, **P < 0.01, Student’s 2-tailed *t* test. Data shown as mean ± SD in all panels where *P* values are shown.

After 11 weeks of treatment, aortic trees and roots were stained with Oil Red O to visualize the lipid distribution. The Oil Red O–positive, lipid-rich aortic areas were significantly smaller in the PRX-treated group than in the control group (*P* = 0.034, Figure 9A). Characterization of atherosclerotic lesions in the aortic sinus of *Ldlr^–/–^* mice treated with PRX or PBS revealed marked reductions in intimal thickening and lipid-rich necrotic cores in subvalvular sections (Figure 9, B and C). Masson’s trichrome staining revealed that PRX treatment also suppressed development of the thick connective tissue deposits that otherwise covered the necrotic cores (Figure 9B).

We also performed flow cytometry to analyze the effects of PRX on cells within the thoracic aorta. The fraction of CD11b^+^Ly6G^–^F4/80^+^ macrophages among live cells (Figure 9D) was significantly smaller in the PRX-treated group than the controls. Moreover, intracellular TNF-α levels were lower in aortic macrophages from PRX-treated mice than from control mice (Figure 9E). Aortic expression of proinflammatory genes, including *Tnf*, *Il1b*, *Ccl5*, *Ccl7*, and *Ccl8*, was lower in the PRX-treated group than the controls (Figure 9F). These findings strongly suggest that PRX suppresses plaque development by suppressing macrophage accumulation and inflammatory activation within the plaques. Notably, PRX also suppressed the accumulation of lipids and macrophages within the liver (Supplemental Figure 8C).

To then test whether 2-hydroxyl-βCD would have an antiatherogenic action in *Ldlr^–/–^* mice, the mice were fed a high-cholesterol diet and treated with the same dose of 2-hydroxypropyl-βCD (1,000 mg/kg body weight, every 2 days) for 11 weeks. βCD treatment did not affect serum lipid profiles over the treatment period (Supplemental Figure 9A), nor did it alter plaque size or morphology in *Ldlr^–/–^* mice (Supplemental Figure 9, B and C).

### Monocyte cholesterol levels correlate positively with human atherosclerosis.

The results summarized so far demonstrate that cellular cholesterol is integral to innate inflammatory activation of mouse macrophages and that disruption of this mechanism using PRX inhibits atherogenesis in *Ldlr*^–/–^ mice. To begin to determine whether this immunometabolic mechanism is also important for human atherosclerosis, we investigated the association between monocyte cholesterol levels and atherosclerosis in human individuals.

**Figure 10:**
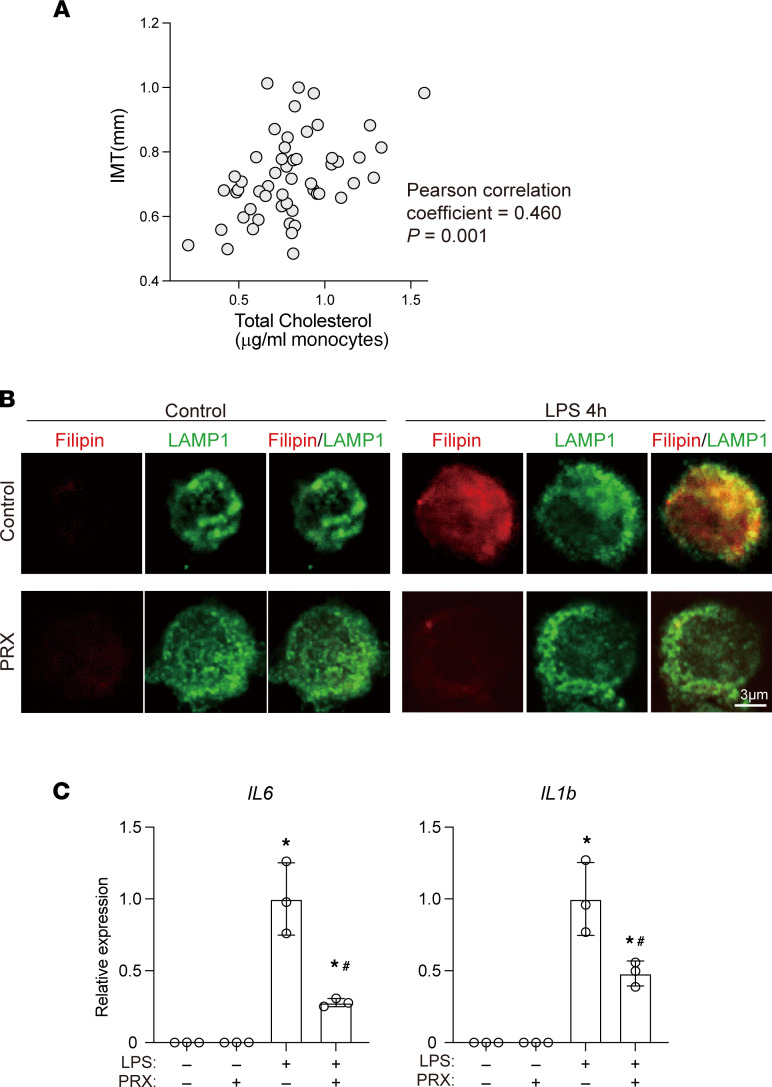
Human monocyte cholesterol and inflammation. (**A**) Monocyte cholesterol levels correlated positively with carotid IMT in humans. (**B**) Human CD14^+^ monocytes were treated for 20 hours with PRX (2 mM βCD) or vehicle prior to the 4-hour LPS stimulation. Localization of free cholesterol (visualized with filipin) and LAMP1 was assessed using confocal microscopy. Scale bar, 3 μm. (**C**) Human CD14^+^ monocytes were treated for 20 hours with PRX prior to 4 hours with LPS. *IL6* and *IL1B* mRNAs were quantified using qPCR. *n* = 3. *P < 0.05 vs. LPS-untreated cells, ^#^*P* < 0.05 vs. LPS only–treated cells, Tukey-Kramer post hoc test. Data shown as mean ± SD in all panels where *P* values are shown.

We first measured cellular cholesterol levels in circulating monocytes and carotid intima-media thickness (IMT) in individuals without known cardiovascular disease. The cholesterol content of the monocytes correlated positively with carotid IMT (Figure 10A), whereas plasma total cholesterol, LDL-cholesterol, HDL-cholesterol, and triglyceride levels did not (Supplemental Figure 10). Multivariate analysis showed that the cholesterol content of monocytes was significantly associated with increased IMT (*P* = 0.001) after adjustment for age, BMI, and plasma HDL-cholesterol, LDL-cholesterol, and triglyceride. This suggests monocyte cholesterol level is a marker of atherosclerotic burden in asymptomatic individuals.

### PRX inhibits inflammatory activation of human monocytes.

The association between monocyte cholesterol and atherosclerosis in humans prompted us to test the antiinflammatory action of PRX on human monocytes to gain insight into its potential as an antiatherosclerotic drug in humans. To that end, CD14^+^ monocytes were collected from peripheral blood and subjected to immunofluorescence imaging. We found that 4 hours of LPS stimulation increased the filipin signal, which at least partially colocalized with the LAMP1-positive area (Figure 10B). As in RAW cells (Figure 2B) and mouse BMDMs (Figure 8A), pretreatment with PRX decreased cholesterol accumulation (Figure 10B). This suggests LPS-induced cholesterol accumulation may also regulate inflammatory activation of human CD14^+^ monocytes. Consistent with that idea, pretreating human monocytes with PRX prior to the LPS stimulation inhibited LPS‑induced expression of *IL6* and *IL1B* mRNA (Figure 10C). These results indicate that PRX suppresses inflammatory activation of human monocytes.

## Discussion

In the present study, we show that activation of cholesterol metabolism and accumulation of cellular cholesterol are required for innate immune responses in macrophages. Our data strongly suggest that LPS activates cholesterol metabolic processes, including its uptake, synthesis, and autophagy/lipophagy in macrophages. They also demonstrate that the accumulation of cholesterol within endolysosomes marks the activation of cholesterol metabolism. This cellular cholesterol accumulation appears to increase the availability of cholesterol to Myd88, which enhances Myd88 self-oligomerization and signal amplification via its CRAC domains. We also show that PRX, which boosts the trafficking of cholesterol from lysosomes and its efflux, suppresses macrophage inflammatory activation in vitro and atherogenesis in vivo. Moreover, the cholesterol level in circulating monocytes is associated with IMT in humans. Collectively, these results indicate that activation of cholesterol metabolism is integral to the innate immune response in macrophages and is an attractive therapeutic target for the treatment of atherosclerosis.

From our results it appears that dynamic activation of cholesterol metabolism is an essential part of programs that control the macrophage response to LPS. Earlier studies have shown that disruption of cholesterol homeostasis enhances TLR4 signaling by modulating lipid rafts (23, 31). LPS causes translocation of CD14, a TLR4 coreceptor, and MAP kinases to lipid rafts in RAW cells. However, Myd88 is not detected in the lipid raft fraction (32). This suggests cholesterol/CRAC domain–mediated Myd88 self‑oligomerization is independent of lipid rafts. Excess accumulation of cholesterol may also activate inflammasomes by forming cholesterol crystals (25, 52). However, treatment with LPS alone is not sufficient to activate inflammasomes (53), which suggests Myd88 signal amplification can be induced by cholesterol independently of inflammasomes or cholesterol crystals.

We found that Myd88 contains 2 conserved CRAC motifs and that cholesterol binding is important for Myd88 self-oligomerization and signal amplification. In several G protein–coupled receptors and ion channels, the CRAC sequence and its mirror sequence, CARC (-[RK]-X(1,5)-Y-X(1,5)-[LV]-), have been identified as cholesterol-binding motifs and have been shown to interact with cholesterol. These motifs were also identified within the cytoplasmic domain of TLRs, close to the transmembrane regions (54). For instance, TLR4 contains 1 CRAC and 2 CARC sequences, which have been proposed to bind cholesterol and to be required for efficient dimerization of TLR4 (54). To the best of our knowledge, however, this model has not yet been directly tested. Although cholesterol binding may also be involved in the activation of TLR4 and other signaling molecules involved in innate immune responses, our finding that PRX inhibits Myd88-dependent TLR4 and TLR9 signaling, but not Myd88‑independent TLR3 and RIG‑I signaling (Supplemental Figure 6D), suggests Myd88 is essential for the enhancement of innate immune signaling by cholesterol and highlights Myd88 as a key molecule linking cellular cholesterol and innate immune signaling.

The effects of interfering with pathways leading to cholesterol accumulation suggest that the increased cellular cholesterol seen after LPS treatment is due to upregulation of cholesterol uptake, synthesis, and autophagy/lipophagy. The findings that mevastatin inhibited both cholesterol accumulation (Figure 2, A and B) and the elevation of cholesterol biosynthesis intermediates (Supplemental Figure 1B) point to the activation of cholesterol synthesis by LPS, though statins may also modulate other processes that can affect cellular cholesterol (35). Chloroquine inhibits autophagy by altering the acidic environment of lysosomes such that it may affect processes other than autophagy (34, 36). Consequently, although our results suggest the involvement of autophagy/lipophagy in LPS-induced cholesterol accumulation, further studies will be needed to clarify the role of autophagy/lipophagy in proinflammatory macrophages. Interestingly, filipin staining appeared as droplets that did not overlap LAMP1 in chloroquine-treated cells (Figure 2B), which suggests that, due to the impaired lipophagy, free cholesterol is localized in organelles other than lysosomes, including lipid droplets (55). Clearly, further studies are needed to elucidate the contribution made by each of the cholesterol metabolic processes to the subcellular distribution of cholesterol and to the innate activation program. In addition, the activation of both cholesterol synthesis and uptake suggests that a mechanism may exist that controls SREBP activity elicited independently of the ER cholesterol level in response to TLR4 activation.

PRX decreases cellular cholesterol and suppresses both inflammatory activation of macrophages and atherogenesis (Figure 9). PRX contains multiple threaded βCDs, which are released within endolysosomes. NPC1 and NPC2 are the key molecules that mediate cholesterol export from lysosomes (56). NPC1 is a transmembrane cholesterol transporter, and NPC2 is a soluble protein that binds cholesterol and transfers it onto NPC1. Together, they act to export cholesterol from late endosomes and lysosomes toward other cell compartments, including the plasma membrane and ER. Although the precise mechanism for the promotion of lysosomal cholesterol export by PRX remains unknown, βCDs released from PRX within endolysosomes may bind to free cholesterol and may provide cholesterol to lysosomal proteins, such as NPC1, and/or the lysosomal membrane (47). In addition, PRX was shown to reduce cellular cholesterol in *NPC1*-deficient cells (44), which suggests PRX also promotes cholesterol efflux independently of NPC1. Our findings indicate that ABCA1-mediated cholesterol export is important for PRX’s effect on cellular cholesterol, as the ABCA1 inhibitor PSC-833 canceled the action of PRX (Figure 6F). This suggests PRX promotes trafficking of cholesterol from lysosomes for ABCA1-mediated efflux.

PRX increased expression of *Abca1* (Figure 6H), which is a target gene of LXR and SREBP1, 2 transcription factors whose activities were enhanced by PRX. PRX increased cellular levels of 2 endogenous LXR ligands, desmosterol and 25‑hydroxycholesterol (Figure 8, C and D) (50, 57). Because it also upregulated SREBP1 target genes and genes related to cholesterol homeostasis (Figure 8, E and F), PRX likely activates the cholesterol biosynthetic pathway at least in part via SREBP1. An earlier study showed that *Npc1* overexpression in CHO cells reduced ER cholesterol levels and upregulated SREBP activity (58). In addition, *Npc1* or *Npc2* mutation impaired production of oxysterols in fibroblasts (59). Those findings, indicating the involvement of NPC1/2 in cholesterol metabolism, are consistent with the effects of PRX. These results support the notion that PRX promotes cholesterol export and trafficking in a fashion similar to NPCs and that PRX activates LXR in part through SREBP1 upregulation. The activation of these transcription factors leads to the upregulation of ABCA1, which promotes cholesterol efflux from macrophages, an effect we would expect to decrease the availability of cholesterol to Myd88.

PRX did not inhibit Myd88-independent TLR3 and RIG-I signaling (Supplemental Figure 6D). In fact, *Il6* induction by the TLR3 ligand poly(I-C) and the RIG-I ligand 3pRNA was somewhat enhanced by PRX. These results are consistent with earlier reports that suppression of Myd88 increases TLR3 activity and that MyD88-knockout mice display an exacerbated inflammatory response following poly(I-C) stimulation (60–62). Those findings further support the notion that PRX inhibits Myd88. That said, because impaired ABCA1-mediated cholesterol efflux was shown to modulate lipid rafts (23, 31), PRX may also affect signaling through lipid rafts and other inflammatory signaling pathways.

Whereas PRX reduced atherogenesis, 2-hydroxypropyl-βCD failed to do so (Supplemental Figure 9). In addition to these superior antiinflammatory actions of PRX over 2-hydroxypropyl-βCD, PRX does not directly interact with membrane cholesterol, enabling it to avoid the intrinsic toxicity of βCDs (44, 46). Because PRX must be taken up by cells to be activated, it may more selectively affect cells with high phagocytotic activity, including macrophages. These characteristics support the notion that PRX is a potentially effective antiinflammatory and antiatherogenic agent. In contrast to our findings, Zimmer et al. reported that βCD suppresses atherogenesis in *Apoe*^–/–^ mice (63). The reason for this difference is not immediately clear. It may be due to differences in the mouse models (*Ldlr*^–/–^ vs. *Apoe*^–/–^) and/or βCD doses used. The actions of βCDs can differ depending on the modifications to the βCD molecule and its dosage as well as the availability of lipoprotein and cholesterol in culture medium (64). Accordingly, the in vivo actions of PRX will need to be further analyzed.

In the present study we demonstrated that cellular cholesterol, particularly cholesterol within endolysosomes, is increased in response to LPS and that this increase is important for TLR4/Myd88 signaling. Lipid-laden foam cells are a hallmark of atherosclerotic plaques. However, recent studies showed that foam cells may be less inflammatory than nonfoamy macrophages (50, 65), though cholesterol crystal formation due to excess cholesterol may also activate inflammasomes (25, 52). Apparently, the effects of cholesterol accumulation within macrophages may differ depending on the cells’ activation state, the timing after stimulation, and the organelles affected. For instance, we showed here that free cholesterol accumulation within endolysosomes was integral to the acute proinflammatory response to LPS. Over a longer time course, however, excess cholesterol is stored as cholesterol ester within foam cells. In the present study, we found that cellular cholesterol was increased by LPS and that PRX-mediated reduction in cholesterol was associated with inhibition of inflammatory activation in both RAW cells and BMDMs. This suggests the accumulation of cellular cholesterol is required for the amplification of TLR4/Myd88 signaling in BMDMs, just as it is in RAW cells. However, earlier studies showed that there are certain differences in the regulatory mechanisms in these cells (66, 67). Moreover, recent studies have revealed that there is broad diversity in the activated states of macrophages and their functions (68, 69). Consequently, the signaling mechanism identified in the present study may contribute differently to the innate response in different populations of macrophages.

In sum, our data demonstrate that cholesterol metabolism is integral to macrophage innate activation. Our findings that decreasing endolysosomal cholesterol with PRX suppressed inflammatory activation of macrophages and atherogenesis and that the cholesterol level in circulating monocytes was associated with the severity of atherosclerosis in humans strongly suggest that macrophage cellular cholesterol metabolism is an attractive diagnostic and therapeutic target for the treatment of inflammatory diseases, including atherosclerosis.

## Methods

### Reagents and antibodies.

LPS, mevastatin, and FITC-CTB were purchased from MilliporeSigma. Chloroquine, PSC-833, and 2‑hydroxypropyl-βCD were purchased from GE Healthcare (now Cytiva), Valspodar, and InvivoGen, respectively. PRX consisting of 2-(2-hydroxyethoxy) ethyl carbamate–modified βCDs as cyclic molecules, Pluronic P123 as an axle polymer, and *N*-triphenylmethyl groups as acid-cleavable stopper molecules were synthesized as described previously (45, 46). The antibodies used were anti-Flag (catalog F1804; MilliporeSigma), anti-GST (PM013; MBL), anti-CFP (D153-3; MBL), anti-Myd88 (sc-74532; Santa Cruz Biotechnology), anti-p65 (sc-372; Santa Cruz Biotechnology), anti-GAPDH (ab181602; Abcam), anti-LAMP1 (sc-20011; Santa Cruz Biotechnology), anti-Rab7 (9367), anti-EE1A (3288; Cell Signaling Technology), anti-PDI (3501; Cell Signaling Technology), anti-Lamin A/C (4777; Cell Signaling Technology), and anti-CTB (SAB4200844; MilliporeSigma).

### Cell culture.

RAW264.7 and HEK293T cells (ATCC) were cultured in DMEM supplemented with 10% FBS (Hyclone). For PSC-833 (Abcam) or PRX treatment, RAW cells were treated with 40 μM PSC-833 or PRX (2 mM βCD) for 20 hours prior to further experimentation. Myd88 Y227F mutant RAW cells were made using a CRISPR/Cas9 system. To collect fractions containing lysosomes and endosomes, OptiPrep Density Gradient medium (MilliporeSigma) was used.

### Statistics.

Comparisons between 2 groups were made using 2-tailed Student’s *t* tests. Differences among more than 2 groups were analyzed using 1-way ANOVA followed by Tukey-Kramer post hoc tests. Values of *P* < 0.05 were considered statistically significant, except in analyses involving RNA-Seq.

### Study approval.

All animal experiments were performed using protocols approved by the Nippon Medical School animal care and use committee. The human atherosclerosis study protocol was approved by the Institute for Atherosclerosis Research Committee on Human Research and meets the standards of the Declaration of Helsinki in its revised version of 1975 and its amendments of 1983, 1989, and 1996 (70).

For full details of all these processes, see Supplemental Methods.

## Author contributions

SH, AT, NY, and YO conceived the project and designed experiments. IM, YO, and ANO analyzed data. SH, AT, NN, HK, YC, TM, MK, TVK, and FK performed experiments. SH, AT, IM, and YO interpreted data and wrote the manuscript.

## Supplementary Material

Supplemental data

## Figures and Tables

**Table 1:**
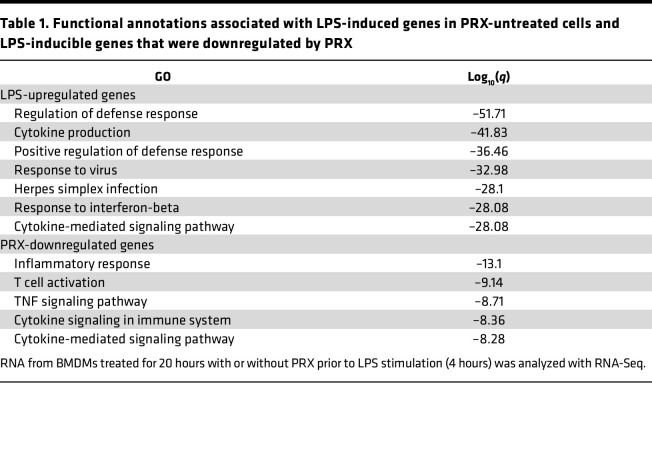
Functional annotations associated with LPS‑induced genes in PRX-untreated cells and LPS-inducible genes that were downregulated by PRX

## Version 1 11/22/2022

Electronic publication
