# Supplementary material for: Activated cholesterol metabolism is integral for innate macrophage responses by amplifying Myd88 signaling
Source: JCI Insight. 2022 Nov 22;7(22):e138539. doi: 10.1172/jci.insight.138539 (PMC9746817; doi:10.1172/jci.insight.138539)
Supplement: Supplemental data [file jciinsight-7-138539-s114.pdf]

SUPPLEMENTAL INFORMATION

Detailed Experimental Procedures

Reagents and antibodies

LPS, mevastatin and FITC-CTB were purchased from Sigma. Chloroquine PSC-833and 2-hydroxypropyl-β-cyclodextrin were purchased from GE Healthcare, Valspodar and InvivoGen, respectively. PRX consisting of 2-(2-hydroxyethoxy) ethyl carbamate-modified β-CDs as cyclic molecules, Pluronic P123 as an axle polymer, and *N*-triphenylmethyl groups as acid-cleavable stopper molecules were synthesized as described previously (1, 2). The antibodies used were anti-Flag (F1804; Sigma), anti-GST (PM013; MBL), anti-CFP (D153-3; MBL), anti-Myd88 (sc-74532; Santa Cruz Biotechnology), anti-p65 (sc-372; Santa Cruz Biotechnology), anti-GAPDH (ab181602; Abcam), anti-LAMP1 (sc-20011; Santa Cruz Biotechnology), anti-Rab7 (#9367), anti-EE1A (#3288), anti-PDI (#3501), anti-Lamin A/C (#4777; Cell Signaling) and anti-CTB (SAB4200844; Sigma).

Cell culture

RAW264.7 and HEK293T cells (ATCC) were cultured in DMEM (Nakalai Tesque) supplemented with 10% heat-inactivated FBS (Hyclone, GE Healthcare) or lipid-deprived FBS (Biowest). BMDM was established as described previously(3).

Generation of Myd88 Y227F mutant RAW cells

Myd88 Y227F mutant RAW cells were made using CRISPR-Cas9 system. The guide RNA was designed to target exon 4 of the mouse *Myd88* genome as follows:

gRNA (Fwd): 5′ - TGACGATTATCTACAGAGCA -3′

gRNA (Rev): 5′ - TGCTCTGTAGATAATCGTCA-3′

The pair of oligonucleotides were ligated into the pSpCas9(BB)-2A-GFP plasmid (Origene PX458), which was then purified using a Midi Plus Ultrapure Plasmid Extraction System (Viogene). A ~2 kb 5’ homology arm and a ~2 kb 3’ homology arm were cloned into a donor plasmid for homologous recombination. Lipofectamine 2000 (Invitrogen) was then used according to the manufacturer's instructions to transfect RAW cells with the plasmids carrying gRNA and the donor plasmids. In parallel, RAW cells transfected with non-silencing plasmid were used as controls. Two days after transfection, cells expressing GFP were selected through fluorescence-activated cell sorting (FACS Aria II cell sorter; BD Bioscience). Sorting of GFP-positive cells was independently

performed at least three times. The single cells obtained were cultured separately, after which a single nucleotide mutation within exon 4 of *Myd88*-Y227F mutant cells was confirmed by DNA sequencing.

Cell imaging

RAW cells, BMDMs, and human monocytes were fixed in paraformaldehyde for 15 min, rinsed with PBS, and incubated for 1 h with 25 μg/ml filipin III (Cayman) in PBS at room temperature in the dark. For immunofluorescent staining, fixed cells were blocked in 5% BSA/PBS, then stained with primary antibodies at 4°C overnight. This was followed by incubation with Alexa fluor-conjugated secondary antibodies (Life technology) for 1 h at room temperature. Cellular images were obtained using a LSM 710 (Zeiss) or BC43 (Oxford instruments) confocal microscope.

Subcellular fractionation

RAW cells incubated with or without LPS for 4 h were subjected to fractionation. To collect fractions containing lysosomes and endosomes, OptiPrep Density Gradient medium (Sigma-Aldrich) was used according to the manufacturer's protocol. The fractions obtained were confirmed by western blotting using antibodies against lysosome-

and endosome-specific proteins.

GC/MS analysis for cholesterol

d7-Cholesterol (0.4 μg/mL in pyridine, 8 μL; Avanti Polar Lipids) was added to suspensions of treated cells or culture medium (1 mL). For extraction of cellular sterols, a chloroform-methanol mixture (chloroform:methanol = 1:2), chloroform and distilled water were successively added to the suspensions. The organic phases were collected and then evaporated to dryness in a nitrogen stream at 40°C. The dried residues were derivatized with *N*-methyl-*N*-(trimethylsilyl)trifluoroacetamide (Sigma-Aldrich) for 30 min at 60°C. The resulting solutions were applied to GC/MS for the quantification of sterols. The GC/MS measurements were made on a GCMS-QP2020 (Shimadzu) equipped with an MXT-1 capillary column (Restek) and used in the selected-ion monitoring mode. The ions used for the quantification and determination of cholesterol, d7-cholesterol, and other sterols were set as described previously (2). The measurement results were normalized by the amount of protein contained in the cell or organelle fraction, which was determined with BCA assays.

Cholesterol efflux assay

RAW cells were cultured for 24 h in DMEM containing with 0.5 μCi/ml of [^{3}H]-cholesterol (PerkinElmer, Waltham, MA) and 1% FBS. After washing the cells twice with serum free medium, DMEM supplemented with 1% mouse serum, as a cholesterol acceptor, was added. Then, PRX (2 mM) and/or PSC-833 (40 μM) were added to the medium, and the cells were cultured for 20 h prior to 4-h LPS stimulation (100 ng/ml). The chase medium was collected and subjected to brief centrifugation to pellet any residual debris. The cleared medium was used to determine the radioactive counts released from the cells. Radioactivity within the cells was determined by extraction in hexane:isopropanol (3:2) followed by solvent evaporation in a scintillation vial prior to counting.

Cholesterol efflux assays using fluorescently labeled cholesterol were carried out using a Cholesterol Efflux Assay kit (Abcam, ab196985) according to the manufacturer's protocol. Briefly, RAW cells were labelled with fluorescently labelled cholesterol for 1 h, followed by culture for 16 h in equilibration medium. The culture medium was then replaced with fresh medium supplemented with 1% mouse serum, and the cells were cultured with or without PRX (2 mM) for 20 hours prior to LPS (100 ng/ml, 4 h) stimulation. Cholesterol efflux was calculated by dividing the fluorescence intensity in the media by the sum of the fluorescence intensities in the media and cells. (The percent

cholesterol efflux was calculated as 100 × (medium dpm) / (medium dpm + cell dpm)).

RNA isolation and qPCR

Total RNA was using NucleoSpin (Macherey Nagel) according to the manufacturer's protocols. StepOnePlus real-time PCR system (Applied Biosystems) was used for qPCR. Target gene expression was normalized to the level of *Gapdh* for RAW cells, BMDMs, and human monocytes or the level of 18s rRNA for mouse aorta.

RNA-sequencing

Poly-A mRNA was extracted from total RNA using a NEBnext poly(A) mRNA magnetic isolation module (New England Biolab), after which RNA-seq libraries were prepared using a NEBNext Ultra RNA Library Prep kit for Illumina according to the manufacturer's protocol (New England Biolab). Libraries were PCR-amplified for ~12 cycles and sequenced on a Hi-seq 1500 (Illumina). Reads were aligned to the mm9 mouse genome using STAR (4). Expression analysis of the RNA-seq data was performed using HOMER (5) and DESeq2 (6). Gene set enrichment analyses were performed using GSEA (7) with rank files generated as previously described (http://genomespot.blogspot.com/2015/01/how-to-generate-rank-file-from-gene.html)

from expression data analyzed using DESeq2. All RNA-seq data are available in the GEO under accession number GSE146236.

Transcription factor target gene identification

To identify SREBP1 target genes, we used our published SREBP ChIP-seq dataset (GSM2095081) and corresponding input data (GSM2095165, GSM2095166) (3). Sequencing data were mapped to the mm10 mouse genome. Peak calling and annotation were performed using HOMER (5). Peaks that overlapped with blacklisted regions and simple repeat regions were removed. HOMER-identified peaks whose scores were ≥30 were selected as high-confidence peaks and used to identify the likely target genes using GREAT (8) with the default parameters.

For identification of LXR target genes, published LXR ChIP-seq and corresponding input data (GSM2095083, GSM2095084, GSM2095087, GSM2095163, GSM2095164) were mapped to the mm10. HOMER (5)-identified peaks whose scores were ≥20 were selected and used to identify target genes using GREAT (8). To further identify genes positively regulated by LXR, RNA-seq data (GSM2095128, GSM2095130, GSM2095179, GSM2095180) were mapped to the mm10. Genes upregulated by the LXR agonist GW3965 were identified using DESeq2 (6). Genes related to LXR peaks and

upregulated by GW3965 were used as the LXR target genes for the analysis in Fig. 6.

Plasmids

Full length cDNAs for mouse wild-type *Myd88* and its CRAC mutants were cloned into pCMV-Flag, pCAGGS-CFP (a generous gift of Dr. T. Taniguchi, University of Tokyo), pGEX-4T-1 (GE Healthcare) vectors to obtain Flag- or CFP-tagged proteins and Myd88-GST fusion protein.

Luciferase reporter analysis

Using Fugene6 (Roche), HEK293T cells were transiently transfected with luciferase reporter plasmids (pNF-kB-Luc and pRenilla-Luc) together with a series of mouse Myd88 expression plasmids. Luciferase activity was measured using a dual-luciferase reporter assay system (Promega).

Cholesterol binding assay

To assess cholesterol binding to Myd88, 1 μg of GST-Myd88 or untagged GST was pre-equilibrated with glutathione resin (Cosmogel GST-Accept) in 50 μl of binding buffer (10 mM Tris-HCl, pH 7.5, 0.15 M NaCl and 0.1% Triton-X). Ten μM ^{3}H-cholesterol

(PerkinElmer) was added and incubated for 30 min at room temperature. The gel was then washed three times with PBS, after which the protein with ^{3}H-cholesterol bound was eluted using 10 mM glutathione and quantified in a liquid scintillation counter (Perkin Elmer) as described earlier (9, 10). For competition experiments unlabeled cholesterol dissolved in ethanol (10 μM, final**)** was added.

To access cholesterol binding to endogenous Myd88, RAW cells were cultured for 24 h in medium containing d7-cholesterol (10 μg/ml) prior to the 4 h of LPS stimulation. Whole cell lysates were collected and subjected to immunoprecipitation with Myd88 antibody, and the amount of d7-cholesterol within the precipitated protein was analyzed with GC/MS.

NBD-cholesterol fluorescence binding assay

22-(*N*-(7-nitrobenz-2-oxa-1,3-diazol-4-yl)-amino)-23, 24-bisnor-5-cholen-3β-ol (NBD-cholesterol; Life Technologies) was added to MyD88 solution (PBS, pH 7.4) to a concentration of 20 nM. The fluorescence intensity of the NBD-cholesterol was then recorded using an FP-8500 fluorophotometer (Jasco, Tokyo, Japan) at 37°C. NBD-cholesterol was excited at 490 nm, and the fluorescence emission spectra were recorded in the range of 510-600 nm. The dissociation constant (K_d) was calculated as

described previously (11).

Myd88 oligomerization assays

To analyze the effects of cholesterol on Myd88 oligomerization, we used GST-Myd88 protein and untagged Myd88. To obtain these proteins, E. coli BL21 were transfected with pGEX-4T-1 vector carrying *Myd88* to produce a recombinant mouse Myd88-GST fusion protein. To obtain native Myd88, Cosmogel GST-Accept (Nakalai Tesque) and a thrombin cleavage capture kit (Merck) were used according to the manufacturer's instructions. The GST-tagged protein complex was recovered and run on SDS-PAGE.

To assess endogenous Myd88 oligomerization, BMDMs were transfected with CFP-tagged and/or flag-tagged Myd88 expression vectors and treated with cholesterol (10 μg/ml in DMSO) for 4 h, as described previously (12). Whole cell lysates were collected and subjected to immunoprecipitation using an anti-CFP antibody. The immunoprecipitated protein was run on SDS-PAGE and blotted with an antibody against Myd88.

Mouse IL-6 ELISA

Culture medium was collected from RAW cells and analyzed using a mouse IL-6 ELISA

kit (R&D) according to the manufacturer's instructions (n=6).

Atherosclerosis study in mice

LDL receptor-deficient (*Ldlr*$^{-/-}$) mice were obtained from Jackson Laboratory (Bar Harbor, ME) and fed a high-cholesterol diet (CRF-1 supplemented with 1.4% cholesterol and 0.4% cholic acid; Oriental Yeast Co.) for 12 weeks beginning at 8 weeks of age. PRX or 2-hydroxypropyl-bCD (Sigma) was dissolved in PBS and sterilized by passage through a 0.2 μm filter. The sterile solution containing PRX or 2-hydroxypropyl-bCD (1000 mg/kg BW) was then administered subcutaneously every other day.

For immunohistochemical analysis, isolated aortic trees and roots were fixed in 4% paraformaldehyde in PBS and then frozen and cut into 6-μm sections. To quantify areas of atherosclerotic lesions, fixed aortic trees and roots were stained with oil red O in 60% isopropanol. Oil red O-positive areas were quantified using ImageJ. Immunohistochemical staining was performed as previously described (13). Briefly, cryosections were fixed in 4% paraformaldehyde/PBS for 10 min at room temperature. The sections were then blocked in 5% BSA/PBS blocking solution, after which primary antibodies prepared in blocking solution were added and incubated overnight at 4°C. After three washes in PBS, the sections were counterstained with Hoechst. Histological

images were captured using an Olympus IX73 microscope.

Flow cytometric analysis

Thoracic aortas were dispersed into individual cells by incubation for 50 min in Dulbecco's phosphate buffered saline (DPBS) containing 400 U/ml type I collagenase (Worthington), 120 U/ml type XI collagenase (Sigma-Aldrich), 60 U/ml hyaluronidase (Sigma-Aldrich), and 60 U/ml DNase I (Sigma-Aldrich) at 37°C. The dispersed cells were pre-incubated with 2.4G2 to block Fc receptors. The antibodies used for the analysis were anti-CD11b (M1/70), anti-CD45 (30-F11), anti-F4/80 (BM8), anti-Ly-6C (HK1.4), anti-Ly-6G (1A8) and anti-TNF-α (MP6-XT22) (all antibodies were from BioLegend). The isotype control for each antibody was also used. For intracellular staining of TNF-α, following surface marker staining, cells were fixed and permeabilized using a Foxp3/Transcription Factor Staining Buffer Set (Thermo). Dead cells were stained by addition of Fixable Viability Dye eFluor™ 780 (Thermo). The samples were analyzed using a FACSVerse (BD Biosciences). Data were analyzed using FlowJo (Tree Star Inc.).

Human atherosclerosis study

To quantify atherosclerotic states in humans, IMT in the common carotid arteries was

measured using ultrasonography in a high-resolution regimen. For this purpose, the distal portions of the right and left carotid arteries were scanned using a lateral angle of interrogation. IMT in the common carotid arteries was measured on the far wall of a 10-mm segment situated just proximal to the carotid sinus. To assess the presence of atherosclerotic plaque, the examination also included a scan of the left and right common carotid arteries, the carotid sinus, and the external and internal carotid arteries in three fixed projections: anterior, lateral and posterior. When visualizing atherosclerotic plaque, carotid arterial stenosis was assessed in a transverse projection. The measurements of IMT and plaque stenosis were carried out using M'Ath computer software (Metris, SRL, France). The average of two measurements (from right and left arteries in lateral position) was considered an integral indicator of mean IMT.

Human monocyte study

After collecting whole blood from healthy donors, monocytes were isolated by centrifuging the blood on a Ficol density gradient followed by magnetic CD14-positive separation using MACS CD14-positive microbeads (Miltenyi Biotec) and MACS separation columns (Miltenyi Biotec). The isolated monocytes were then seeded into sterile 24-well culture plates at a density of 10^6 cells/well and incubated in RPMI

containing 10% FBS without colony-stimulating factors. The cells were cultured for 7 days at 37°C in a humidified CO_2-incubator (95% air and 5% CO_2). On day 7, the medium was replaced with serum-free X-VIVO medium for 1 day, after which 2 mM PRX was added. After an additional 24 h, LPS was added to a final concentration of 100 ng/ml.

siRNA-mediated knockdown

For siRNA-mediated gene knockdown, chemically synthesized siRNA targeting genes of interest and control siRNA (siPerfect Negative Control) were purchased from Sigma. RAW cells were transfected with 50 nM siRNA using Lipofectamine RNAiMAX reagent (Thermo) according to the manufacturer’s protocol. Forty-eight hours after transfection, the cells were used for experimentation.

TLR3, TLR9, and RIG-I activation

RAW cells were stimulated with poly(I:C), CpGB or 3pRNA (at a final concentration of 100 ng/ml) using Lipofectamine 2000 according to the manufacturer's instructions for TLR3, TLR9 and RIG-I activation.

High-performance liquid chromatography

For lipoprotein (CM, VLDL, LDL and HDL) distribution analyses, plasma samples from 4 or 5 mice per group were analyzed using an upgraded high-performance liquid chromatography (HPLC) technique as previously described (Skylight Biotech)(14).

Statistical analysis

Sample sizes were not based on power calculations. No animals were excluded from analyses. Comparisons between two groups were made using two-tailed Student's *t* tests. Differences among more than two groups were analyzed using one-way ANOVA followed by Tukey-Kramer's post-hoc tests. Values of $P<0.05$ were considered statistically significant, except in analyses involving RNA-seq. All data are shown as means ± SEM, except when otherwise noted.

References

1. Tamura A, Nishida K, and Yui N. Lysosomal pH-inducible supramolecular dissociation of polyrotaxanes possessing acid-labile N-triphenylmethyl end groups and their therapeutic potential for Niemann-Pick type C disease. *Sci Technol Adv Mater.* 2016;17(1):361-74.
2. Tamura A, and Yui N. Polyrotaxane-based systemic delivery of beta-cyclodextrins for potentiating therapeutic efficacy in a mouse model of Niemann-Pick type C disease. *J Control Release.* 2018;269:148-58.
3. Oishi Y, Spann NJ, Link VM, Muse ED, Strid T, Edillor C, et al. SREBP1 Contributes to Resolution of Pro-inflammatory TLR4 Signaling by Reprogramming Fatty Acid Metabolism. *Cell Metab.* 2017;25(2):412-27.
4. Dobin A, Davis CA, Schlesinger F, Drenkow J, Zaleski C, Jha S, et al. STAR: ultrafast universal RNA-seq aligner. *Bioinformatics.* 2013;29(1):15-21.
5. Heinz S, Benner C, Spann N, Bertolino E, Lin YC, Laslo P, et al. Simple combinations of lineage-determining transcription factors prime cis-regulatory elements required for macrophage and B cell identities. *Mol Cell.* 2010;38(4):576-89.
6. Love MI, Huber W, and Anders S. Moderated estimation of fold change and dispersion for RNA-seq data with DESeq2. *Genome Biol.* 2014;15(12):550.
7. Subramanian A, Tamayo P, Mootha VK, Mukherjee S, Ebert BL, Gillette MA, et al. Gene set enrichment analysis: a knowledge-based approach for interpreting genome-wide expression profiles. *Proc Natl Acad Sci U S A.* 2005;102(43):15545-50.
8. McLean CY, Bristor D, Hiller M, Clarke SL, Schaar BT, Lowe CB, et al. GREAT improves functional interpretation of cis-regulatory regions. *Nat Biotechnol.* 2010;28(5):495-501.
9. Wang MK, Ren T, Liu H, Lim SY, Lee K, Honko A, et al. Critical role for cholesterol in Lassa fever virus entry identified by a novel small molecule inhibitor targeting the viral receptor LAMP1. *PLoS Pathog.* 2018;14(9):e1007322.
10. Zhang Y, Lee KM, Kinch LN, Clark L, Grishin NV, Rosenbaum DM, et al. Direct Demonstration That Loop1 of Scap Binds to Loop7: A CRUCIAL EVENT IN CHOLESTEROL HOMEOSTASIS. *J Biol Chem.* 2016;291(24):12888-96.
11. Avdulov NA, Chochina SV, Igbavboa U, Warden CS, Schroeder F, and Wood WG. Lipid binding to sterol carrier protein-2 is inhibited by ethanol. *Biochim Biophys Acta.* 1999;1437(1):37-45.
12. Xu H, Xia H, Zhou S, Tang Q, and Bi F. Cholesterol activates the Wnt/PCP-YAP

signaling in SOAT1-targeted treatment of colon cancer. *Cell Death Discov.* 2021;7(1):38.

13. Liu L, Koike H, Ono T, Hayashi S, Kudo F, Kaneda A, et al. Identification of a KLF5-dependent program and drug development for skeletal muscle atrophy. *Proc Natl Acad Sci U S A.* 2021;118(35).
14. Karasawa T, Takahashi A, Saito R, Sekiya M, Igarashi M, Iwasaki H, et al. Sterol regulatory element-binding protein-1 determines plasma remnant lipoproteins and accelerates atherosclerosis in low-density lipoprotein receptor-deficient mice. *Arterioscler Thromb Vasc Biol.* 2011;31(8):1788-95.
15. Humphries WHt, Szymanski CJ, and Payne CK. Endo-lysosomal vesicles positive for Rab7 and LAMP1 are terminal vesicles for the transport of dextran. *PLoS One.* 2011;6(10):e26626.
16. Cheng XT, Xie YX, Zhou B, Huang N, Farfel-Becker T, and Sheng ZH. Characterization of LAMP1-labeled nondegradative lysosomal and endocytic compartments in neurons. *J Cell Biol.* 2018;217(9):3127-39.

Supplemental figure legends

Supplemental Figure 1. Effects of LPS and IL-4 on the cholesterol biosynthetic pathway

A) Scheme of the cholesterol biosynthetic pathway.

B) Using gas-chromatography/mass spectrometry (GC/MS), cholesterol and its biosynthetic intermediates were quantified in RAW cells treated for 4 h with LPS (100 ng/ml). n=4. ***P*<0.01, Student's two-tailed *t*-test.

C, D) RAW cells were treated with mouse recombinant IL-4 (10 nM, Biolegend) for 4 h. In C, expression of *Arg1* mRNA, a marker for IL-4-induced M2 polarization (30). In D, cholesterol and the intermediates were analyzed with GC/MS. n=4. **P*<0.05, ns: not significant, Student's two-tailed *t*-test.

Supplemental Figure 2. Colocalization of the filipin signal with endolysosomes

Confocal microscopic analysis of colocalization of the filipin signal with markers for selected organelles [EEA1 (early endosomes), Rab7 (late endosomes), LAMP1 (lysosomes) and PDI (ER)] in RAW cells before or after LPS stimulation for 4 h. Shown are fractions of the indicated marker-positive area within the filipin$^+$ area. Colocalization was analyzed using Imaris software. Note that, as previously reported, our data suggest

that the EEA1 and Rab7 signals partially overlap the LAMP1 signal (15, 16).

Supplemental Figure 3. Plasma membrane lipid rafts

RAW cells were cultured for 4 h in the presence or absence of LPS, fixed and stained with FITC-CTB. Representative images from three independent experiments are shown.

Supplemental Figure 4. Effects of *Abca1* and *Abcg1* knockdown on RAW cells

A-C) RAW cells were transfected with siRNA targeting *Abca1* and/or *Abcg1* or with control siRNA. Forty-eight hours after transfection, the cells were treated with LPS for an additional 4 h. In A, expression levels of Abca1 and Abcg1 were analyzed by qPCR. In B, the cells were fixed, and cellular cholesterol was visualized using filipin staining. The fluorescence intensity was measured using a luminometer. The obtained values were normalized by the value of unstimulated cells transfected with control siRNA. n=4-6 in each group. *P<0.05, **P<0.01, Tukey-Kramer's post-hoc test. In C, expression levels of *Il6* and *Il1b* were analyzed by qPCR in cells transfected with siRNA targeting *Abca1* and/or *Abcg1*. n=3 in each group. *P<0.05 vs. control siRNA-transfected cells not stimulated with LPS, $^{\#}P$<0.05, ns; not significant. Tukey-Kramer's post-hoc test.

Supplemental Figure 5. CRISPR/Cas9-mediated strategy to generate Myd88 mutant cells

A) Schematic showing the targeting of a Y227F homologous donor to the *Myd88* locus. The mouse *Myd88* locus on chromosome 9 is depicted at the top. The Y227F donor includes the tyrosine-to-phenylalanine mutation (red) in exon 4.

B) Protein expression of Myd88 in control and Myd88 (Y227F) cells was analyzed by western blotting.

Supplemental Figure 6. Effects of PRX on macrophage activation

A) RAW cells were treated for 20 h with Alexa 488-PRX (2 mM) followed by LPS for 4 h. Colocalization of PRX with late endosomes (Rab7) and lysosomes (LAMP1) was visualized using confocal microscopy. Scale bar; 5 mm.

B) Representative images of RAW cells treated with PRX (4 mM) or 2-hydroxyl-bCD (4 mM) for 20 h. Lipid raft was visualized with FITC-CTB. Scale bar; 5 μm.

C) PRX inhibited nuclear translocation of NF-κB p65 subunit. Immunofluorescent staining of NF-κB p65 in RAW cells treated with or without PRX for 20 h followed by treatment with LPS for 4 h, as in Fig. 4B.

D) Relative expression of *Il6* mRNA in RAW cells treated with or without PRX (2 mM, 20 h) followed by treatment with LPS, CpGB, Poly(I:C) or 3PRNA (100 ng/ml) for 4 h. $^{*}P<0.05$ vs. pattern recognition receptor ligand-untreated cells, $^{\#}P<0.05$ vs. PRX-untreated cells, Tukey-Kramer’s post-hoc test.

Supplemental Figure 7. PRX suppresses LPS-inducible genes in BMDMs

A) Heatmaps showing expression levels of 2,026 LPS-inducible genes in cells treated with vehicle, PRX (2 mM) or 2-hydroxypropyl-bCD (2 mM) at the indicated times.

B) Relative expression of mRNAs in BMDMs treated with or without PRX (2 mM) for 20 h followed by treatment with LPS for 4 h. n=3 in each group. Data shown are representative of two independent experiments. Bars show means ± s.d. *P<0.05 vs. LPS-untreated cells, #P<0.05 vs. PRX-untreated cells, Tukey-Kramer’s post-hoc test.

Supplemental Figure 8. Effects of PRX on *Ldlr*$^{-/-}$ mice

A) Body weight changes during the 11-week course of PRX treatment. No significant differences between groups were observed during the test period. Data are means ± s.d. n = 11 mice for each time point; two-way ANOVA and Šídák's multiple comparisons test.

B) Plasma total cholesterol, chylomicron, VLDL, LDL and HDL cholesterol levels in

Ldlr$^{-/-}$ mice treated for 11 weeks with PBS or PRX (n=3, each group). ns: not significant. Student's two-tailed *t*-test.

C) Representative images of sections of liver and spleen from *Ldlr*$^{-/-}$ mice treated for 11 weeks with PBS or PRX. Sections were stained with hematoxylin and eosin (HE) or oil red-O or immunostained for F4/80. Scale bars; 100 μm.

Supplementary Figure 9. Effects of 2-hydroxyl-bCD on *Ldlr*$^{-/-}$ mice

A) Plasma total cholesterol, chylomicron, VLDL, LDL and HDL cholesterol levels in *Ldlr*$^{-/-}$ mice treated for 11 weeks with PBS or 2-hydroxyl-bCD (1000 mg/kg BW) (n=4-5, each group). ns: not significant, Student's two-tailed *t*-test.

B) Representative photographs of aortic sinuses from *Ldlr*$^{-/-}$ mice treated with PBS or 2-hydroxyl-bCD as shown in (A). Sections were analyzed with hematoxylin-eosin (HE) or Masson-Trichrome staining or immunostaining for F4/80. Scale bar: 200 μm.

C) Areas of atherosclerotic lesions, necrotic cores and F4/80 positivity in -hydroxyl-bCD-treated mice were quantified and normalized to those in control mice. Bar graphs represent the mean ± s.d. n=4-5 in each group, ns; not significant. Student's two-tailed *t* test.

Supplementary Figure 10. Human monocyte cholesterol and inflammation

A) Monocyte cholesterol levels correlated positively with carotid IMT in humans.

B) Plasma triglyceride, HDL and LDL levels did not correlate with carotid IMT in the subjects.

C) Human $CD14^+$ monocytes were treated for 20 h with PRX (2 mM) or vehicle prior to the 4-h LPS stimulation. Localization of free cholesterol (visualized with filipin) and LAMP1 were assessed using confocal microscopy. Scale bar, 3 mm.

C) Human $CD14^+$ monocytes were treated for 20 h with PRX prior to 4 h with LPS. *Il6* and *Il1b* mRNAs were quantified using qPCR. n= 3. $^*P<0.05$ vs. LPS-untreated cells, $^{\#}P<0.05$ vs. LPS only-treated cells, Tukey-Kramer's post-hoc test.

Supplementary Table 1: Primers used for qRT-PCR

(Mouse Genes)

**Gene symbol** (Description)	**Forward primer**	**Reverse primer**
Gapdh	AATGTGTCCGTCGTGGATCT	CATCGAAGGTGGAAGAGTGG
18s	GCAATTATTCCCCATGAACG	GGGACTTAATCAACGCAAGC
Il6	ATGGATGCTACCAAACTGGAT	TGAAGGACTCTGGCTTTGTCT
Il1b	TGGGCCTCAAAGGAAAGAAT	CAGGCTTGTGCTCTGCTTGT
Tnf	CAGGCGGTGCCTATGTCTC	CGATCACCCCGAAGTTCAGTAG
Abca1	GCTTGTTGGCCTCAGTTAAGG	GTAGCTCAGGCGTACAGAGAT
Abcg1	GTGGATGAGGTTGAGACAGACC	CCTCGGGTACAGAGTAGGAAAG
Abcb1a	CAGCAGTCAGTGTGCTTACAA	ATGGCTCTTTTATCGGCCTCA
Cxcl9	TTCTTTTCCTCTTGGGCATC	TGAGGGATTTGTAGTGGATCG
Il12b	TGGTTTGCCATCGTTTTGCTG	ACAGGTGAGGTTCACTGTTTCT
Cxcl11	GGCTTCCTTATGTTCAAACAGGG	GCCGTTACTCGGGTAAATTACA
Ccl9	CCCTCTCCTTCCTCATTCTTACA	AGTCTTGAAAGCCCATGTGAAA
Ccl5	GCTGCTTTGCCTACCTCTCC	TCGAGTGACAAACACGACTGC
Ccl7	GCTGCTTTCAGCATCCAAGTG	CCAGGGACACCGACTACTG
Ccl8	TCTACGCAGTGCTTCTTTGCC	AAGGGGGATCTTCAGCTTTAGTA

(Human Genes)

Gene symbol (Description)	Forward primer	Reverse primer
GAPDH	GGAGCGAGATCCCTCCAAAAT	GGCTGTTGTCATACTTCTCATGG
IL6	ACTCACCTCTTCAGAACGAATTG	CCATCTTTGGAAGGTTCAGGTTG
TNF	CCTCTCTCTAATCAGCCCTCTG	GAGGACCTGGGAGTAGATGAG

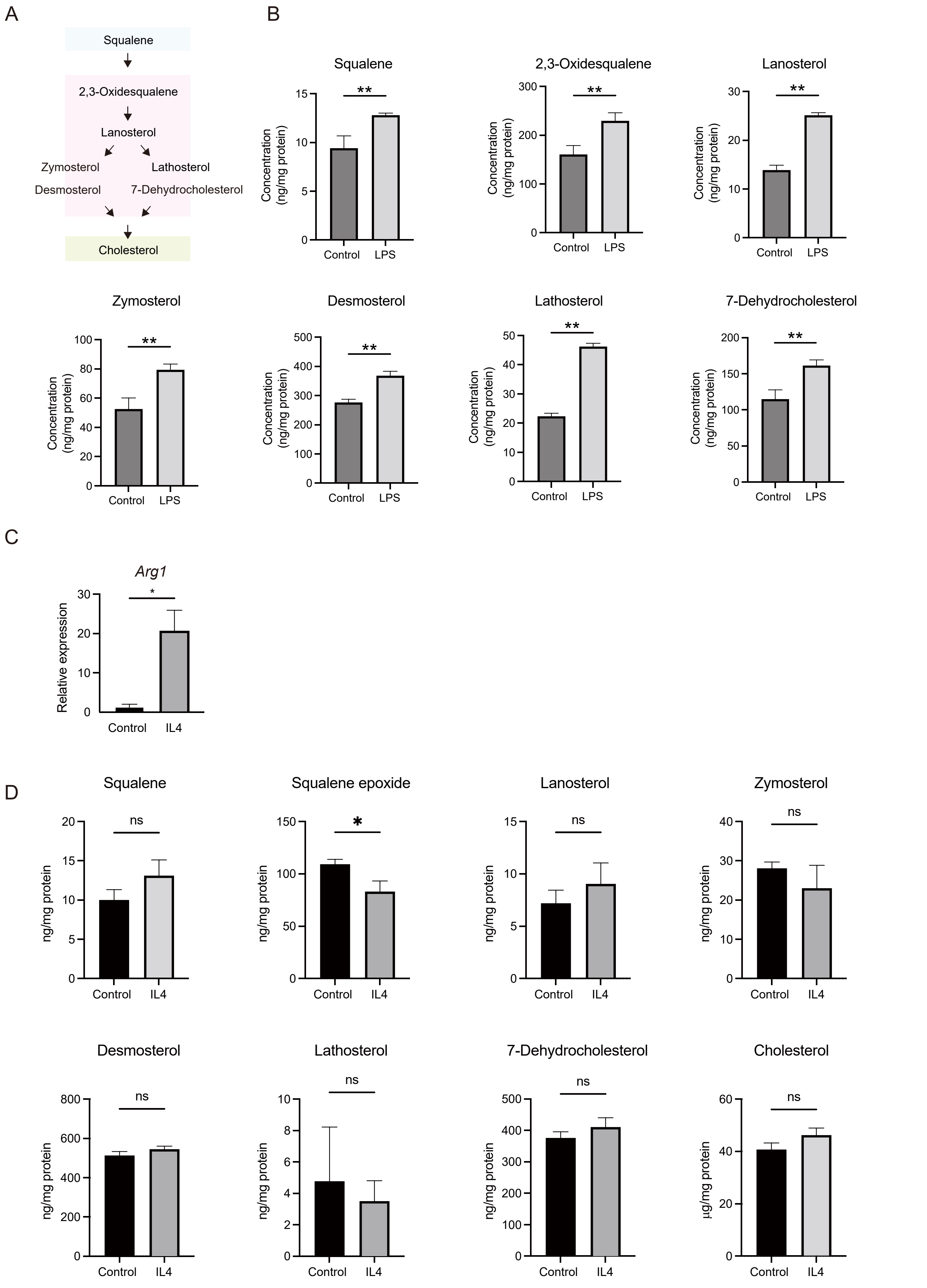
A
Squalene
2,3-Oxidesqualene
Lanosterol
Zymosterol
Lathosterol
Desmosterol
7-Dehydrocholesterol
Cholesterol
B
Squalene
2,3-Oxidesqualene
Lanosterol
Zymosterol
Desmosterol
Lathosterol
7-Dehydrocholesterol
Concentration (ng/mg protein)
Control
LPS
**
C
Arg1
Relative expression
Control
IL4
*
D
Squalene
Squalene epoxide
Lanosterol
Zymosterol
Desmosterol
Lathosterol
7-Dehydrocholesterol
Cholesterol
ng/mg protein
µg/mg protein
ns
*
Control
IL4

Suppl. Fig. 1

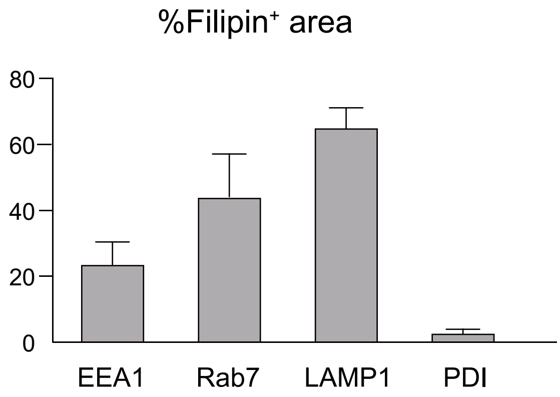

Suppl. Fig. 2

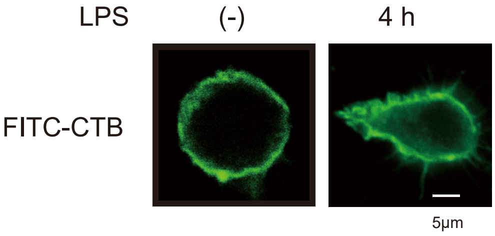

Suppl. Fig. 3

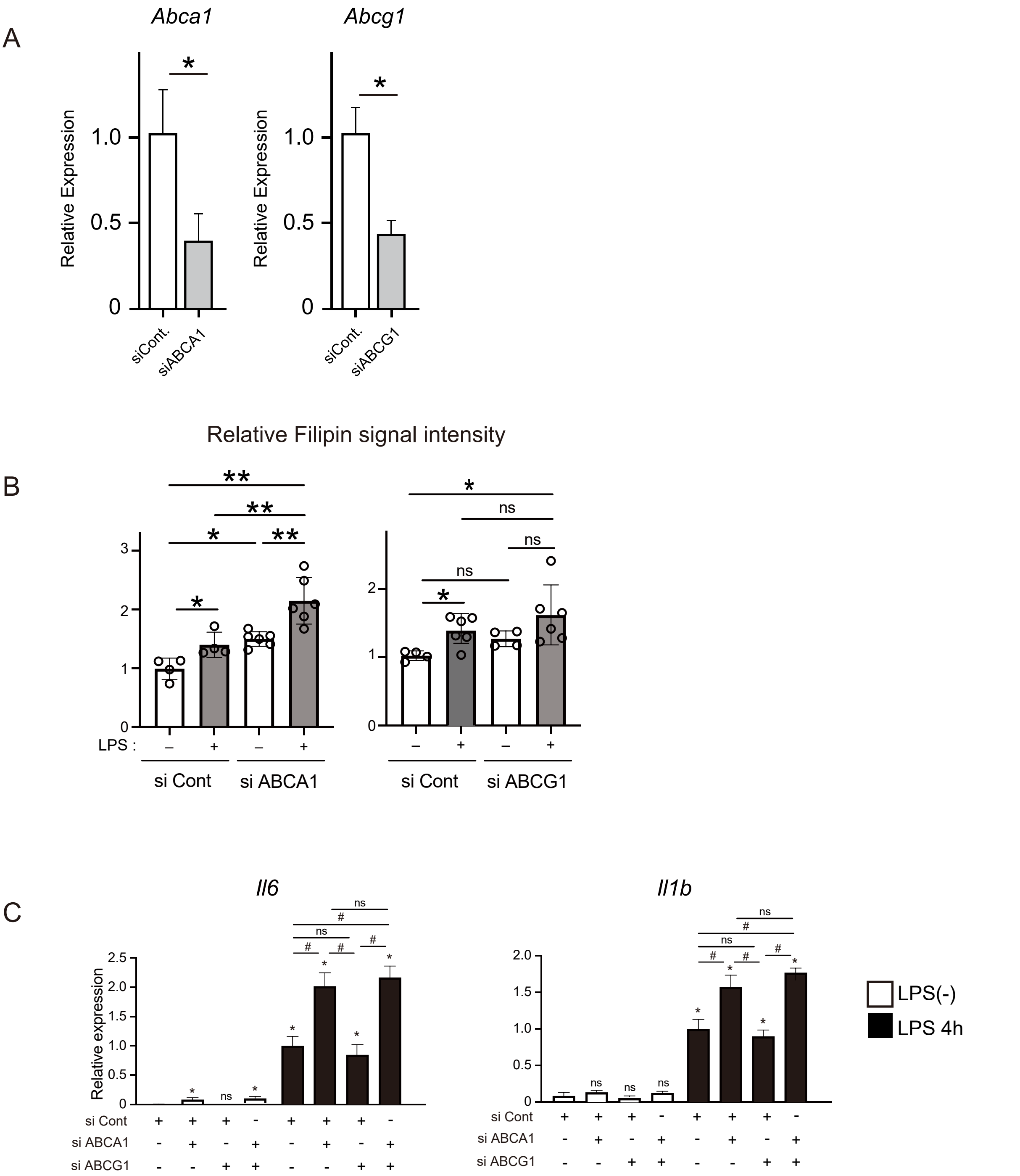
A
Abca1
Abcg1
Relative Expression
1.0
0.5
0
siCont.
siABCA1
siABCG1
Relative Filipin signal intensity
B
**
*
ns
LPS :
si Cont
si ABCA1
si ABCG1
C
Il6
Il1b
Relative expression
#
si Cont
si ABCA1
si ABCG1
LPS(-)
LPS 4h

A

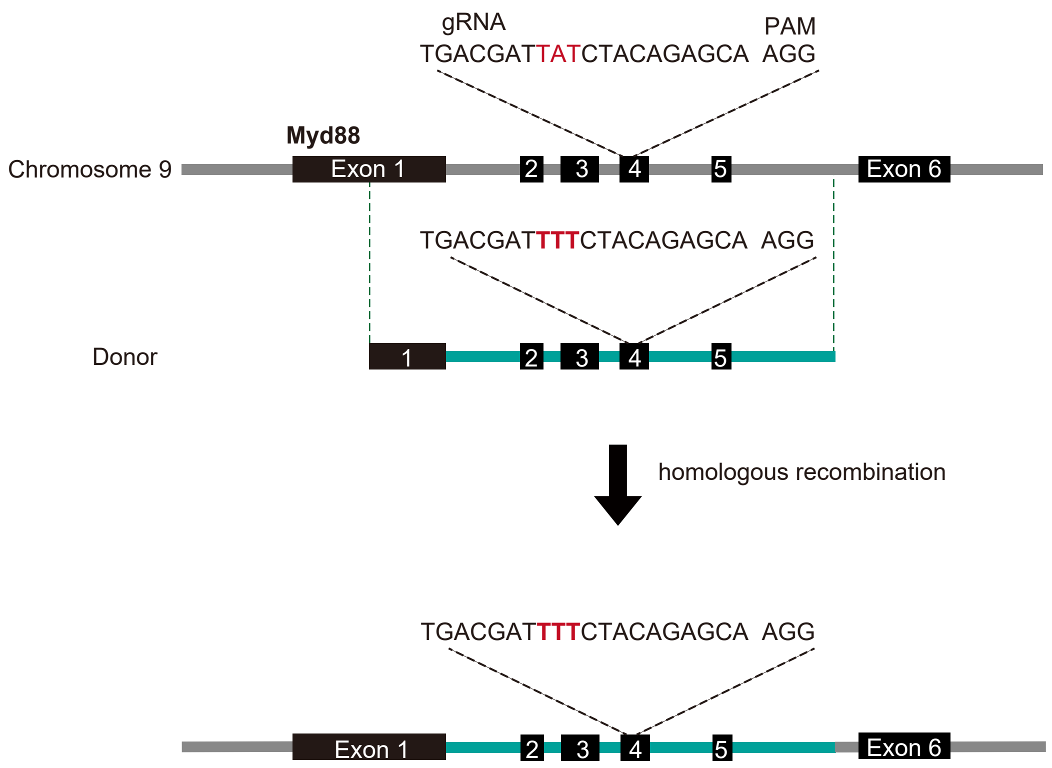
gRNA
PAM
TGACGATTATCTACAGAGCA AGG
Myd88
Chromosome 9
Exon 1
2
3
4
5
Exon 6
TGACGATTTTCTACAGAGCA AGG
Donor
1
2
3
4
5
homologous recombination
TGACGATTTTCTACAGAGCA AGG
Exon 1
2
3
4
5
Exon 6

B

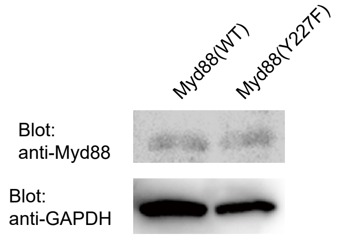
Myd88(WT)
Myd88(Y227F)
Blot:
anti-Myd88
Blot:
anti-GAPDH

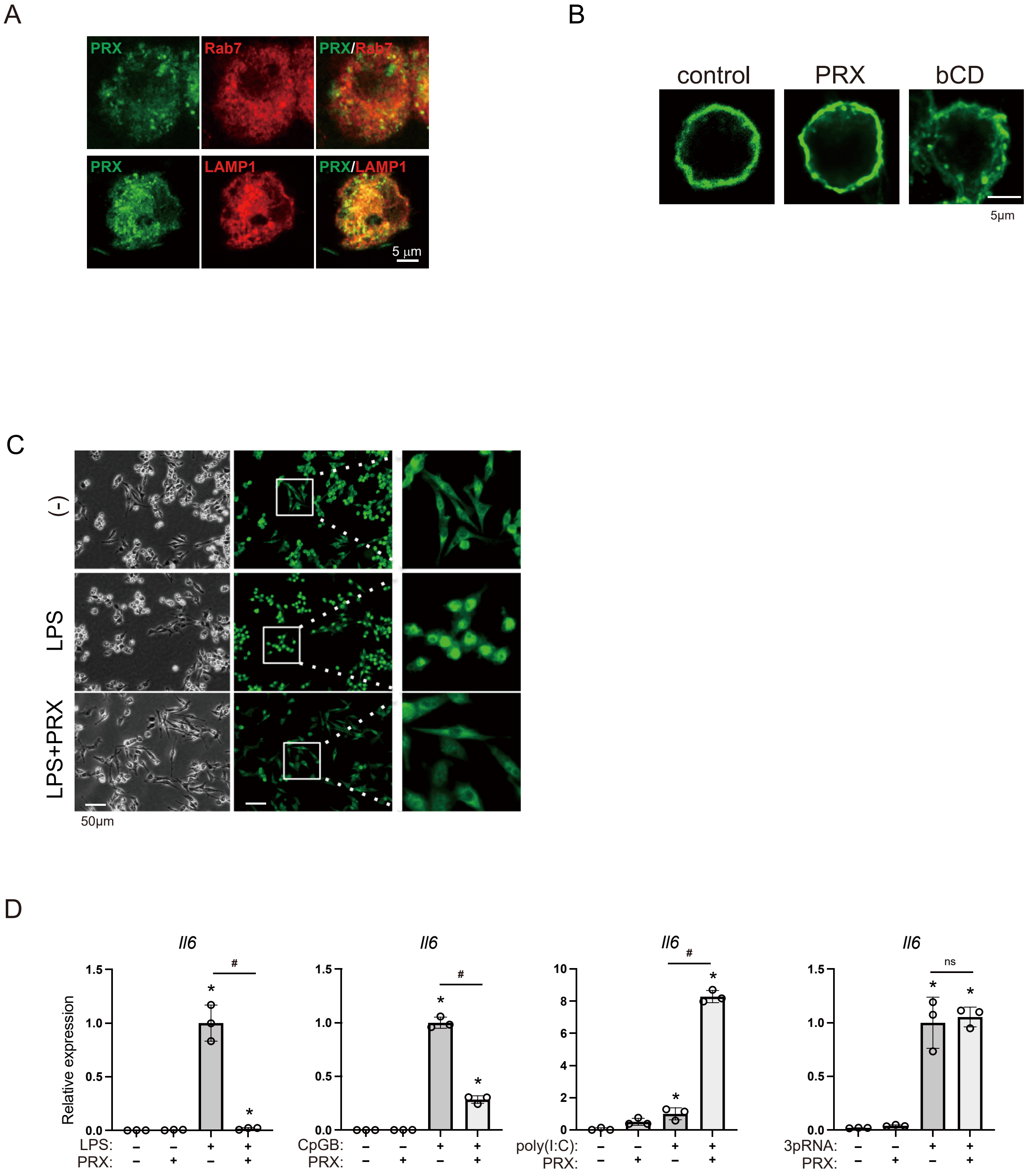
A
PRX
Rab7
PRX/Rab7
PRX
LAMP1
PRX/LAMP1
5 µm
B
control
PRX
bCD
5µm
C
(-)
LPS
LPS+PRX
50µm
D
Il6
Relative expression
LPS:
PRX:
CpGB:
poly(I:C):
3pRNA:
#
ns
*

Suppl. Fig. 6

A

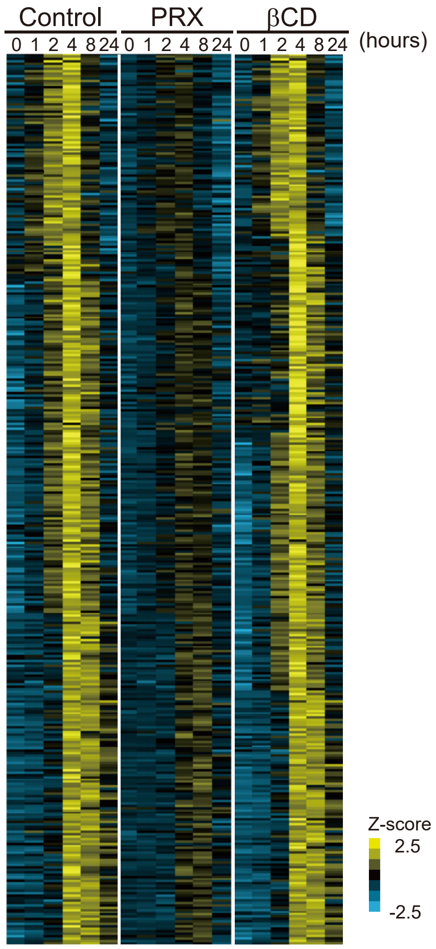

B

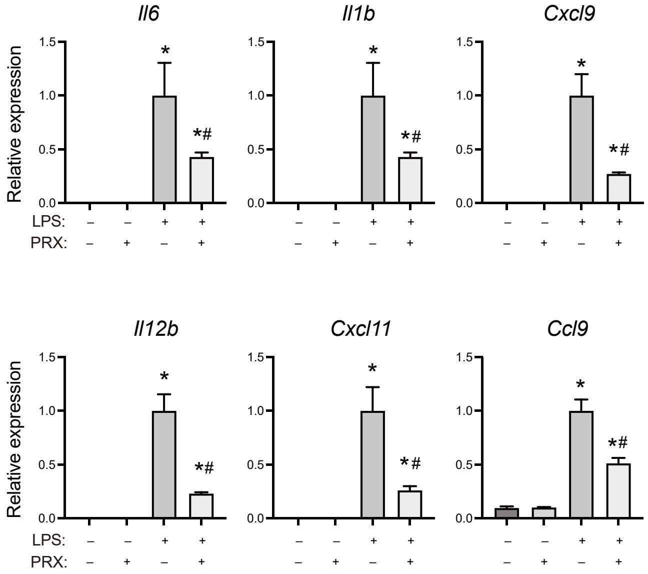

Suppl. Fig. 7

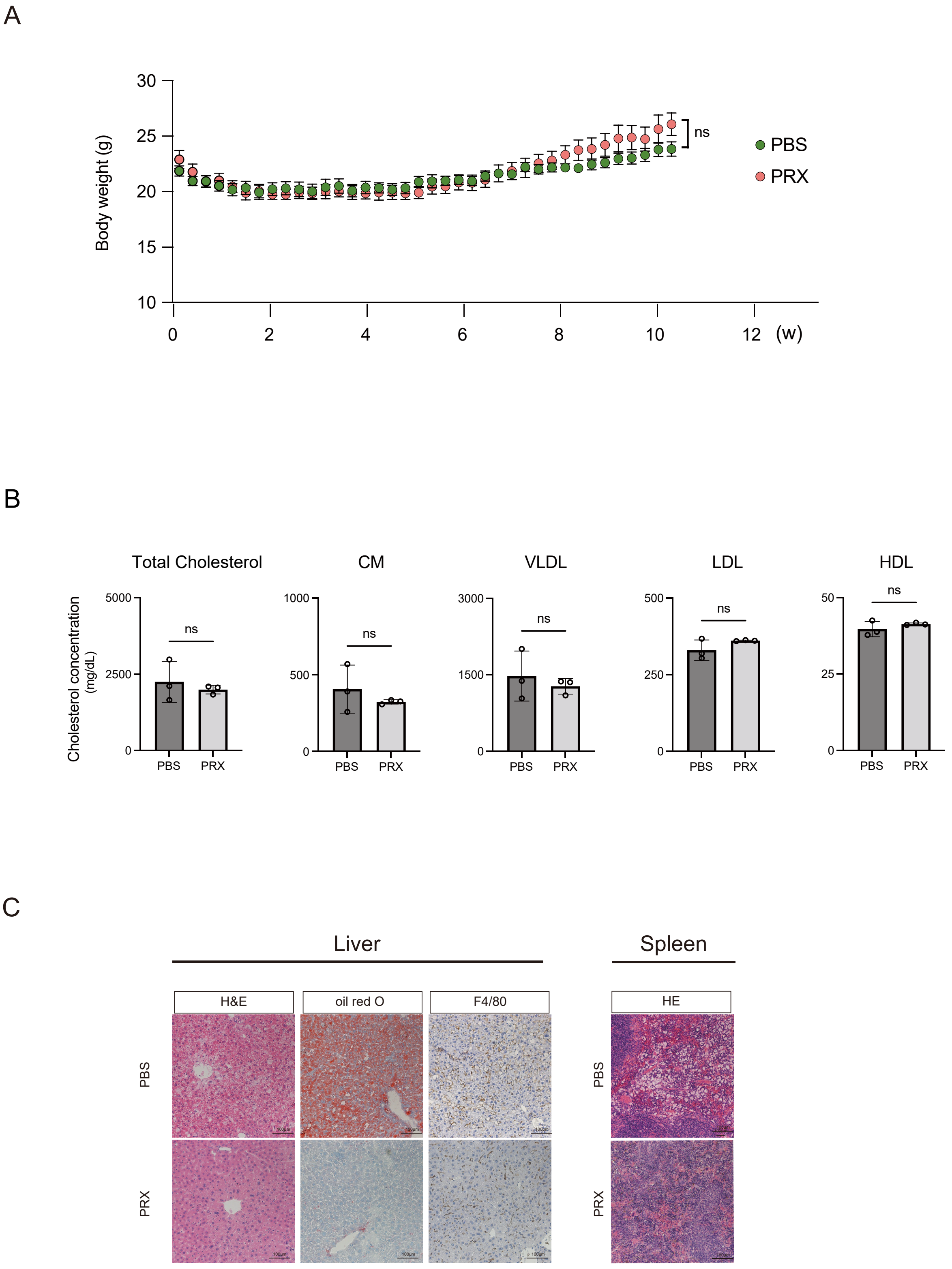
A
Body weight (g)
30
25
20
15
10
0
2
4
6
8
10
12
(w)
ns
PBS
PRX
B
Total Cholesterol
CM
VLDL
LDL
HDL
Cholesterol concentration (mg/dL)
5000
2500
0
1000
500
3000
1500
250
50
25
ns
PBS
PRX
C
Liver
Spleen
H&E
oil red O
F4/80
HE
PBS
PRX

Suppl. Fig. 8

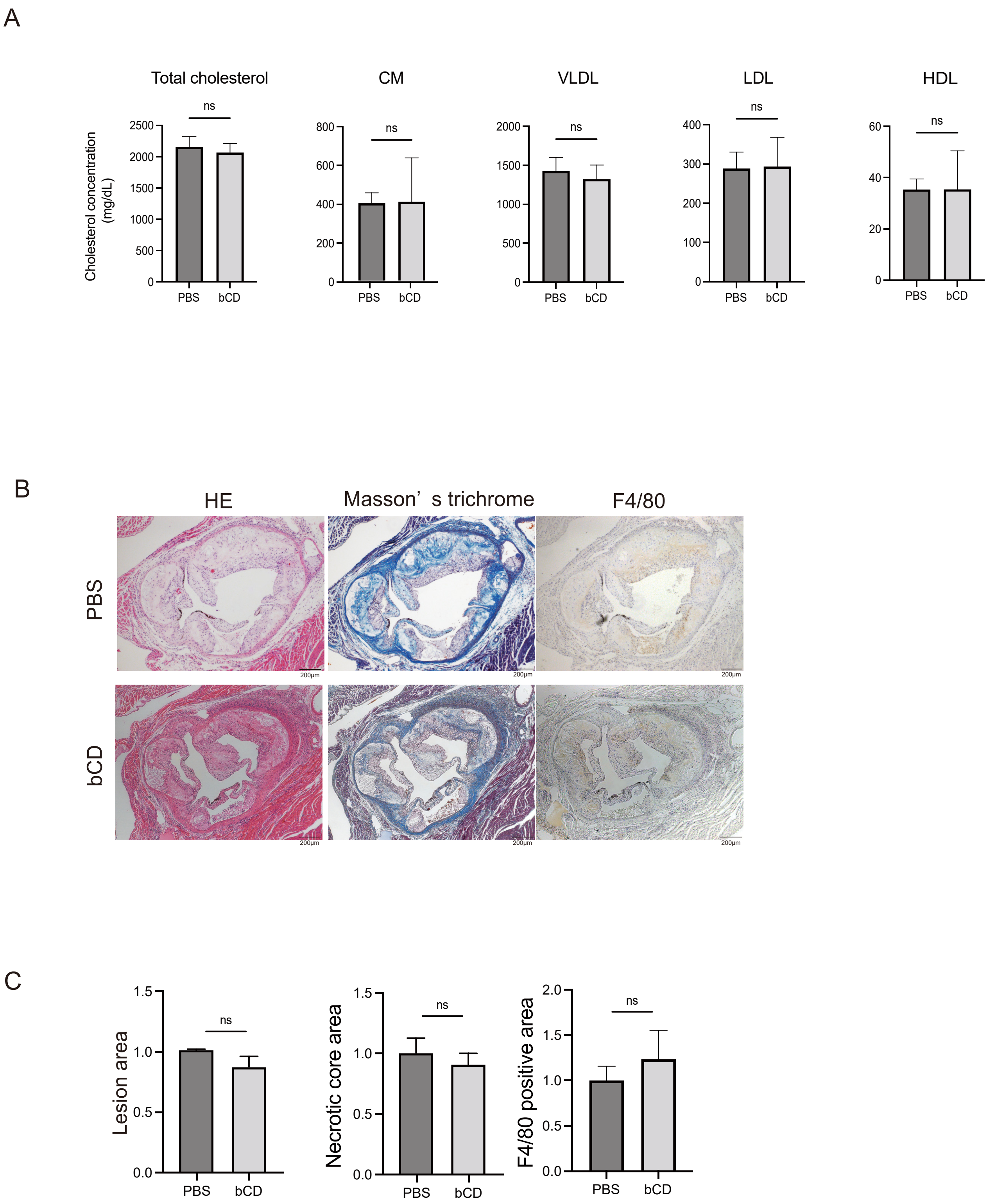
A
Total cholesterol
CM
VLDL
LDL
HDL
Cholesterol concentration (mg/dL)
ns
PBS
bCD
B
HE
Masson’ s trichrome
F4/80
PBS
bCD
200μm
C
Lesion area
Necrotic core area
F4/80 positive area
ns
PBS
bCD

Suppl. Fig. 9

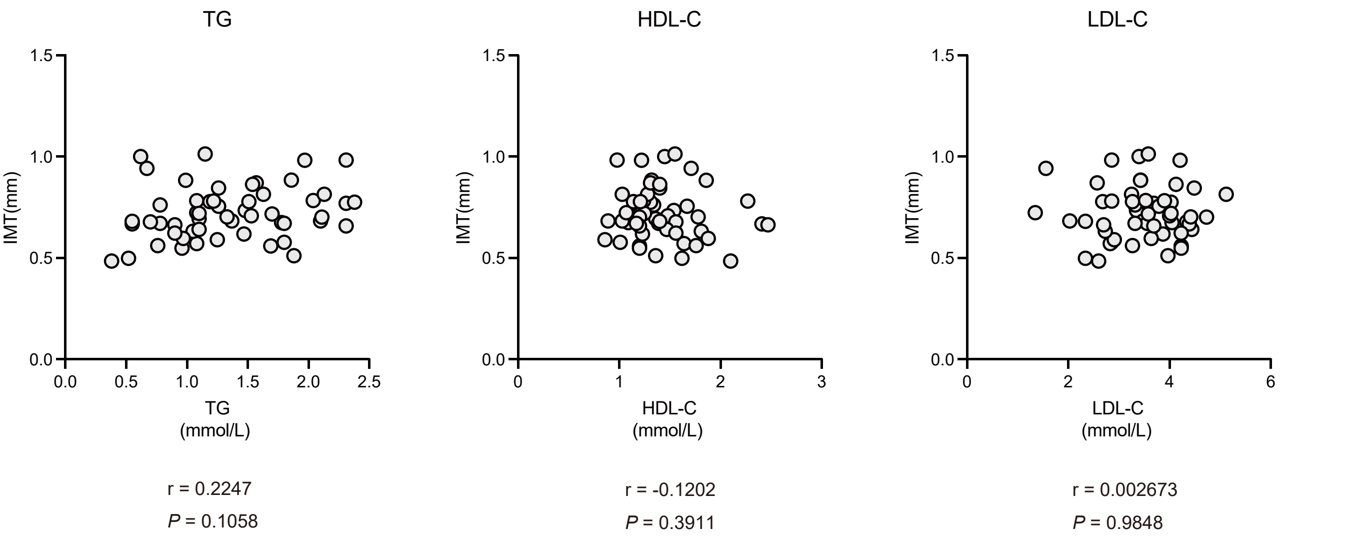
TG
HDL-C
LDL-C
IMT(mm)
TG
(mmol/L)
HDL-C
(mmol/L)
LDL-C
(mmol/L)
r = 0.2247
P = 0.1058
r = -0.1202
P = 0.3911
r = 0.002673
P = 0.9848

Suppl. Fig. 10
